# VIRMA/IGF2BP3-mediated ANLN upregulation promotes intrahepatic cholangiocarcinoma growth by forming a positive feedback loop with RhoA/YAP1/TEAD1 signaling pathway

**DOI:** 10.1038/s41419-025-08197-5

**Published:** 2026-01-09

**Authors:** Jiajun Zhang, Ning Huang, Lin-rui Gao, Ai Guo, Hongming Deng, Liming Wang, Mei Liu

**Affiliations:** 1 Department of Hepatobiliary Surgery, National Cancer Center/National Clinical Research Center for Cancer/Cancer Hospital, Chinese Academy of Medical Sciences and Peking Union Medical College, Beijing, China; 2 Laboratory of Cell and Molecular Biology & State Key Laboratory of Molecular Oncology, National Cancer Center/National Clinical Research Center for Cancer/Cancer Hospital, Chinese Academy of Medical Sciences and Peking Union Medical College, Beijing, China

**Keywords:** Oncogenes, Cell signalling

## Abstract

The prognosis of patients with intrahepatic cholangiocarcinoma (ICC) remains poor owing to the lack of effective targeted therapeutic strategies. Thus, the exploration of the molecular pathogenesis of ICC is urgently required. The cytoskeleton protein, anillin (ANLN), has been reported to contribute to various tumor growth by participating in cytokinesis via RhoA signaling. However, the exact physiological role and potential regulatory mechanism of ANLN in ICC are still not well understood. Based on spindle-related genes, integrated bioinformatic analyses identified ANLN as a potential candidate target for ICC. ANLN was elevated in ICC and predicted worse survival. Mechanistically, VIRMA-mediated m6A modification and IGF2BP3-dependent interaction collectively accounted for the upregulation of ANLN by maintaining its mRNA stability. Furthermore, the combination of ANLN and VIRMA or IGF2BP3 offered a greater predictive value than each marker alone in a large ICC cohort. Functional studies indicated that ANLN was involved in cancer cell proliferation and cell cycle. ANLN knockdown induced cytokinesis failure, DNA damage, and apoptosis in ICC cells. In addition to discovering the crucial role of ANLN in cytokinesis via RhoA activation, we also illustrated that ANLN restrained the Hippo pathway by enhancing the activity of RhoA signaling, which together contributed to ANLN-mediated tumor-promoting effects on ICC. Furthermore, YAP1-TEAD1 transcriptionally activated ANLN, subsequently establishing a self-reinforcing loop between ANLN and Hippo pathway, which was mediated by RhoA signaling as an intermediate regulatory node. Importantly, two clinical drugs, the RhoA inhibitor simvastatin and the YAP1/TEAD inhibitor verteporfin were determined to be the disruptors of this feed-forward signaling axis, inhibiting ICC tumor growth. These findings reveal the vital function of ANLN in ICC growth and provide promising treatment strategies for ICC.

## Introduction

Intrahepatic cholangiocarcinoma (ICC), the second most frequently diagnosed liver malignancy, accounts for up to 20% of all hepatic malignancies, with a rising morbidity [1]. Due to the lack of specific and sensitive diagnostic markers, ICC is frequently diagnosed at its advanced stages, making surgical intervention ineligible [2]. Advanced ICC patients are less likely to benefit from chemotherapy and immunotherapy [3]. In addition, the molecular pathogenesis of ICC is poorly investigated. Thus, identifying the crucial genes that play significant roles in the onset and advancement of ICC will offer a theoretical framework for uncovering new therapeutic targets.

Recently, a high-throughput drug screening study has reported that spindle protein inhibitors have potent tumor-suppressive effects in various preclinical cholangiocarcinoma models [4]. Inducing chromosomal instability by targeting mitotic processes can exceed a critical threshold, causing cell cycle arrest and subsequent induction of apoptosis. This approach has been proposed as a promising strategy for the treatment of liver cancer [5, 6]. Therefore, we hypothesized that inhibition of mitotic spindle-related genes may be an effective anticancer therapeutic approach for ICC treatment. However, the significance and underlying regulatory mechanisms of ICC development are poorly elucidated.

In this study, based on a set of mitotic spindle-related genes, integrative bioinformatic analysis determined anillin (ANLN) as a potential candidate contributing to ICC growth and progression. In biological research, ANLN is a widely known mitotic protein, playing an essential role in cytokinesis by recruiting the actomyosin contractile ring, and has been reported as an oncoprotein [7, 8]. The transformation of inactive RhoA-GDP to active RhoA-GTP, is essential for the initiation of cytokinesis and the assembly of actomyosin contractile ring [9, 10]. Various researches have indicated that ANLN ensures cytokinesis via triggering RhoA activation, which relies on the interaction between ANLN and RhoA regulatory kinases, including ECT2, PLK1 and RACGAP1 [8, 11–13]. As for oncology research, ANLN is remarkably upregulated in numerous malignancies and strongly related to poor prognosis [12, 14, 15]. Furthermore, ANLN has been implicated in various cancer-related activities such as cell growth, infiltration, maintenance of stem cell properties and cytokinesis [14–17]. Yet, the exact physiological role and potential regulatory pathways of ANLN in ICC are still not well investigated.

The Hippo pathway is evolutionarily conserved and regulates tissue size and differentiation via the transcriptional co-activator YAP1 and the transcription factors TEAD1-4 [18, 19]. Studies have increasingly shown the critical importance of the Hippo pathway in driving oncogenic activities, including cellular growth and metastasis [19–21]. The Hippo pathway was reported to participate in cytokinesis [5]. Furthermore, an increase in YAP1 activity has been observed in cholangiocarcinoma [21]. RhoA GTPase (active RhoA) is an upstream regulator to inactivate the Hippo pathway and promote YAP1 activation via cascaded dephosphorylation of LATS1 [5, 18, 22, 23]. Simultaneously, previous researches have demonstrated that RhoA physically interacts with the anillin homology domain of ANLN and that its activity is regulated by ANLN via induction of the RACGAP1 phosphorylation mediated by PLK1 and ECT2 [11–13, 24]. These results suggest that ANLN might inhibit the Hippo pathway and activate YAP1 activity via RhoA signaling.

N6-methyladenosine (m6A) is the most abundant post-transcriptional alteration, controlled by m6A methyltransferase writers (such as METTL3, METTL14, METTL16, VIRMA, and RBM15) and demethylase eraser (such as ALKBH5 and FTO). Specific RNA-binding proteins (such as YTHDF1/2/3, IGF2BP1/2/3, and YTHDC1/2) act as m6A readers to recognize the m6A motif of target genes, thus affecting their mRNA stability, translation, and splicing [25]. Existing evidences have illustrated the essential role of m6A modification in tumor progression and treatment resistance [26, 27]. However, the functions of m6A modifications in ICC have not been thoroughly investigated.

In this study, we aimed to delineate the role of ANLN in ICC and the potential regulatory mechanisms of epigenetic modification. Our study increases the understanding of the molecular mechanism of ANLN and provides a biomarker for ICC therapy.

## Results

### Integrative bioinformatic analysis indicates ANLN as a potential candidate involving in ICC progression

**Fig. 1:**
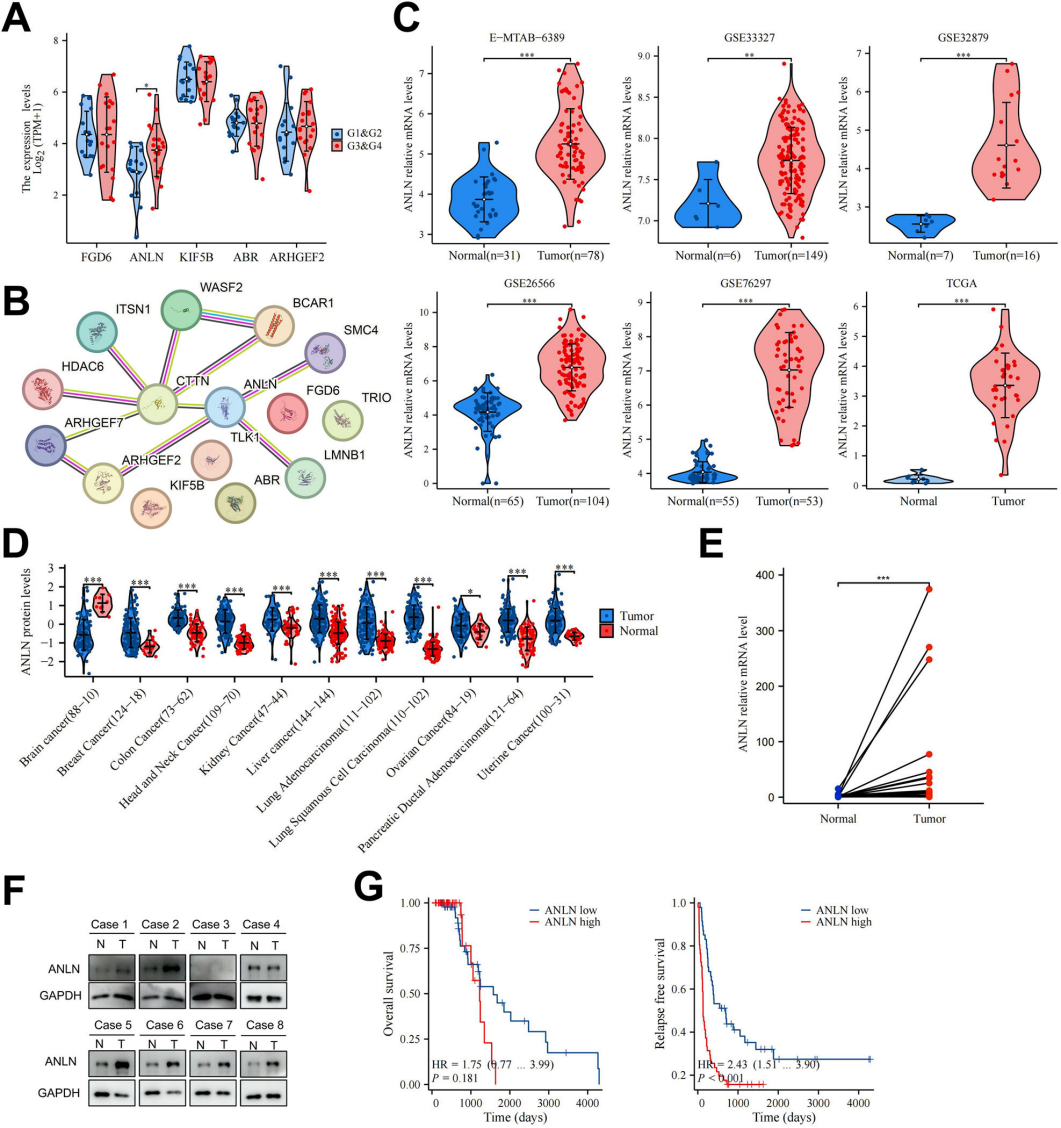
The expression of ANLN is elevated in ICC and related with patient prognosis. **A** The top 5 upregulated genes mRNA level in different pathological grades. **B** The protein interaction of 15 DEGs predicted in STRING database. **C** The mRNA levels of ANLN in cholangiocarcinoma and non-tumor tissues of six data sets (E-MTAB-6389, GSE33327, GSE32879, GSE26566, GSE76297, TCGA). **D** The protein levels of ANLN in pan-cancer tissues and adjacent non-tumor tissues from cProSite proteogenomic database. **E** The mRNA expression of ANLN in 25 paired tumor tissues of ICC and their corresponding adjacent non-tumor tissues. **F** Western blotting of ANLN expression in eight paired ICC tumor tissues and non-tumor tissues. N, non-tumor tissue; T, tumor tissue. **G** The overall survival (left) and recurrence free survival (right) of 98 patients diagnosed with ICC were performed in ANLN high group (*n* = 51) and ANLN low group(*n* = 47). These results are presented as mean values with standard deviation (SD). * *p* < 0.05, ** *p* < 0.01, *** *p* < 0.001.

In this study, 199 mitotic spindle-related genes were downloaded from the MSigDB database. In addition, three ICC transcriptome datasets, including E-MTAB-6389, GSE32879, and GSE33327, were acquired with differentially expressed genes (DEGs) preformed (adjusted *p*-value < 0.05) (Supplementary Fig. S1A). Of the 199 genes associated with spindles, 15 DEGs were intersected in all three ICC datasets (Supplementary Fig. S1B). The heat map illustrated the expression levels of these genes arranged according to their median rank across the analyses in three datasets. The five most significant DEGs identified were FGD6, ANLN, KIF5B, ABR, and ARHGEF2 (Supplementary Fig. S1C). Among these five DEGs, ANLN was related to tumor grade (Fig. 1A). To screen the key regulators among these 15 DEGs, we constructed a protein-protein interaction network in the STRING database. ANLN, ARHGEF2, ARHGEF7, CTTN, and BCAR1 were potential regulators (Fig. 1B). Based on the above bioinformatic analysis, we initially identified ANLN as the most potent candidate. The box plots showed the significant upregulation of ANLN in the tumor tissues of six cholangiocarcinoma datasets (Fig. 1C). Besides, ANLN protein was notably increased in ten types of tumors analyzed using the online cProSite proteogenomic data (Fig. 1D). For further verification, ANLN mRNA and protein levels were assessed in ICC tumors and corresponding non-tumor adjacent tissues. The findings showed an increase in ANLN expression in ICC tumor specimens (Fig. 1E, F).

In a 98-ICC-patient cohort, patients were distinguished as high group and low group according to ANLN levels (Supplementary Fig. S1D). For the clinicopathological characteristic, the ANLN expression level is related with increased probability perineural invasion (*p* = 0.0736) (Supplementary Fig. S2A; Supplementary Table S1). In this cohort, univariate analyses indicated that better OS is related with female and worse RFS is related to increased tumor size, microvascular invasion, perineural invasion, lymph node metastasis and elevated ANLN expression (Supplementary Fig. S2B, C). The multivariate analyses illustrated that better OS is related with female and decreased tumor size, and worse RFS is related with increased tumor size, lymph node metastasis, and elevated ANLN expression (Supplementary Fig. S2D, E). Thus, our study demonstrated that ANLN is elevated in ICC and suggests poor prognosis, especially RFS.

### VIRMA-mediated m6A modification of ANLN maintains its IGF2BP3-dependent mRNA stability

**Fig. 2:**
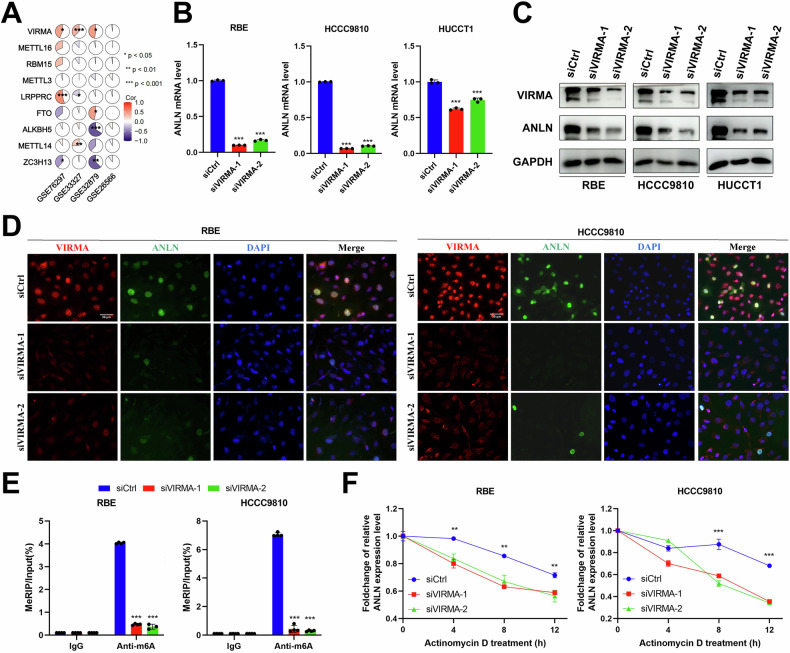
VIRMA-mediated m6A modification of ANLN mRNA maintains its stability. **A** The expression correlations between m6A writers or erasers and ANLN in four cholangiocarcinoma data sets. **B** The levels of mRNA for ANLN in RBE, HCCC9810, and HUCCT1 cells that were transfected with either siVIRMA or siCtrl. **C** The levels of protein for ANLN in RBE, HCCC9810, and HUCCT1 cells that were transfected with either siVIRMA or siCtrl. **D** RBE and HCCC9810 cells that were transfected with either siVIRMA or siCtrl underwent dual immunofluorescence staining. The representative images were presented. Scale bar=30μm. **E** RBE and HCCC9810 cells underwent transfection with either siVIRMA or siCtrl, were followed by immunoprecipitation to assess the levels of m6A-modified mRNA in 4044 site of 3′UTR, which was evaluated using qPCR analysis. **F** The RBE and HCCC9810 cells, which were transfected with either siVIRMA or siCtrl, were treated with actinomycin D (5 μM) and incubated for time periods of 0, 4, 8, and 12 h. The measurement of ANLN mRNA levels were carried out using qPCR, with the results being normalized to the 0 h reference point. These results are presented as mean values with standard deviation (SD). *n* = 3–4. * *p* < 0.05, ** *p* < 0.01, *** *p* < 0.001.

Based on the aforementioned findings, we firstly aimed to clarify the regulatory mechanism that governs the upregulation of ANLN expression in ICC. Initially, ANLN genomic amplification or mutation was infrequently observed in multiple ICC and HCC datasets (<3%) according to the cBioPortal genomics database (Supplementary Fig. S3A). Thus, the genomic variations in ANLN are unable to explain its upregulation in ICC. Given the elevated levels of ANLN mRNA, we primarily aimed to explore the transcriptional regulation or post-transcriptional regulation mechanisms of ANLN in ICC. Recently, dysregulation of m6A regulators has been reported to be involved in the regulation of target gene expression via epigenetic regulatory mechanisms in human cancer [25]. In addition, the SRAMP database revealed potential multiple methylation modification sites in the ANLN mRNA sequence (Supplementary Fig. S3B, C). Therefore, we aimed to investigate potential post-transcriptional regulators of ANLN in ICC cells via m6A modification. Based on the expression profiles of four cholangiocarcinoma data sets, the correlations analysis between ANLN and m6A writers and erasers were performed. The results indicated a marked positive correlation between VIRMA and ANLN in the three datasets, whereas other m6A writers or erasers did not (Fig. 2A and Supplementary Fig. S4A). Thus, VIRMA was speculated to be a potential candidate for regulating ANLN in an m6A dependent way. Consistent with our hypothesis, VIRMA knockdown remarkably reduced ANLN mRNA and protein expression in ICC cells (Fig. 2B–D). A former research effort demonstrated that VIRMA primarily facilitated mRNA methylation within the 3′UTR or close to the stop codon [28]. Thus, specific primer were designed for the potential 4044 m6A site in 3′UTR predicted by SRAMP database (Supplementary Fig. S3B, C). The data from MeRIP-qPCR indicated that the enrichment of ANLN mRNA mediated by the m6A-specific antibody, was considerably reduced in ICC cells lacking VIRMA (Fig. 2E). When RBE and HCCC9810 cells were treated with Actinomycin D, the results suggested that VIRMA affected the stability of ANLN mRNA (Fig. 2F).

**Fig. 3:**
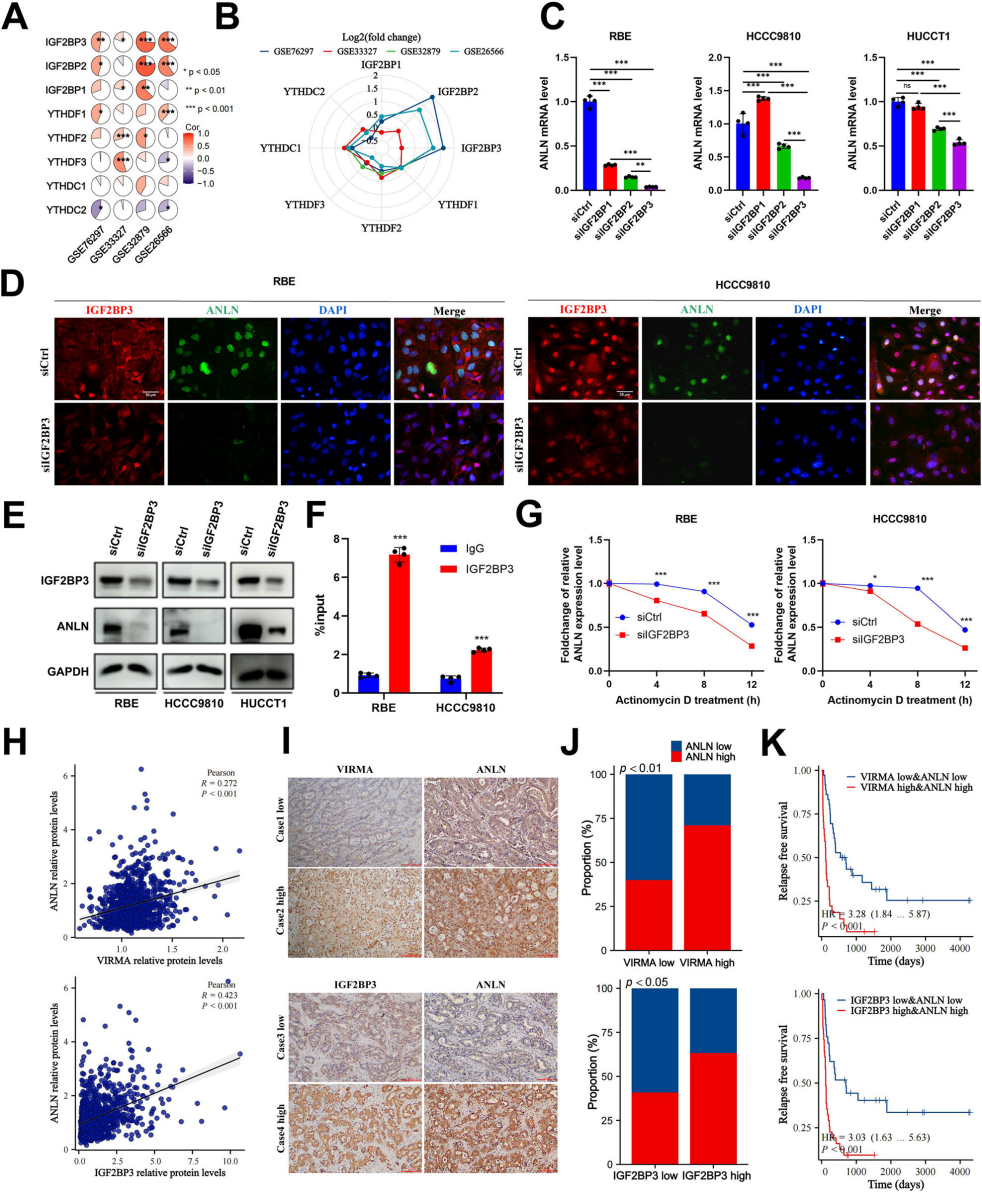
IGF2BP3 acts as the most essential m6A reader to maintain the stability of m6A-modified ANLN mRNA. **A** The expression correlations between m6A readers and ANLN in four cholangiocarcinoma data sets. **B** The radar map for the expression levels of m6A readers in cholangiocarcinoma datasets. The mRNA levels of these genes were analyzed using log2 transformation to calculate the fold change between tumor tissues and their adjacent non-tumor counterparts. **C** The mRNA levels of ANLN in RBE, HCCC9810 and HUCCT1 cells transfected with siIGF2BP1/2/3 or siCtrl. **D** RBE and HCCC9810 cells, which were transfected with either siCtrl or siIGF2BP3, underwent dual immunofluorescence staining. The displayed images were representative. Scale bar=30μm. **E** The levels of the ANLN protein in RBE, HCCC9810, and HUCCT1 cells transfected with either siIGF2BP3 or siCtrl by western blotting. **F** RIP assay was performed by utilizing anti-IGF2BP3 antibodies or control IgG. RNA was extracted from magnetic beads and subjected to qPCR analysis. **G** RBE and HCCC9810 cells transfected with siIGF2BP3 or siCtrl were subjected to incubation with actinomycin D (5 μM) for time intervals of 0, 4, 8, and 12 h. The quantification of ANLN mRNA level was performed using qPCR, with the measurements normalized to the 0 h time point. **H** Pearson correlation analysis of VIRMA or IGF2BP3 protein level and ANLN protein level in pan-cancer tissues from cProSite proteogenomic database. **I** Representative images showing staining of VIRMA, IGF2BP3, and ANLN in samples from ICC patients. Scale bar = 100μm. **J** The relationship between ANLN level and the expression of VIRMA or IGF2BP3 in ICC tissues was assessed with a chi-square test. **K** A comparison of RFS was conducted between ICC patients with low levels of ANLN, VIRMA, or IGF2BP3 and those with high levels of ANLN, VIRMA, or IGF2BP3 using Cox regression analysis. These results are presented as mean values with standard deviation (SD). *n* = 3–4. * *p* < 0.05, ** *p* < 0.01, *** *p* < 0.001.

To accurately determine the m6A reader that recognizes the m6A motif of ANLN, correlation analysis was performed. The results revealed that ANLN was positively correlated with IGF2BP1/2/3 and YTHDF1/2/3 (Fig. 3A). Meanwhile, we found that IGF2BP1/2/3 had higher expression levels than YTHDF1/2/3 in cholangiocarcinoma (Fig. 3B). Therefore, we investigated the effects of IGF2BP1/2/3 on ANLN mRNA stability. Strikingly, IGF2BP3 knockdown showed the most obvious inhibitory effects at the ANLN mRNA level, compared to IGF2BP1 or IGF2BP2 knockdown (Fig. 3C). Scatter diagrams showed significant positive correlations between IGF2BP3 and ANLN expression at the mRNA level (Supplementary Fig. S4B). Furthermore, immunofluorescence staining and western blotting exhibited that IGF2BP3 knockdown reduced ANLN protein level (Fig. 3D, E). RNA immunoprecipitation assays illustrated the binding of IGF2BP3 with ANLN mRNA in ICC cells (Fig. 3F). Furthermore, a decrease in ANLN mRNA stability was observed following IGF2BP3 knockdown (Fig. 3G).

Additionally, IHC staining was conducted to detect VIRMA and IGF2BP3 proteins in the same cohort. The levels of VIRMA and IGF2BP3 were elevated in a variety of tumors, including ICC (Supplementary Fig. S5A–C). Moreover, significant positive correlations between VIRMA or IGF2BP3 protein expression and ANLN protein expression were observed in human pan-cancers (Fig. 3H). In our study, both VIRMA and IGF2BP3 protein levels were positively correlated with ANLN expression in ICC tissue samples (Fig. 3I, J). In our investigation, the elevated expression levels of ANLN (Fig. 1G), VIRMA (Supplementary Fig. S5D), and IGF2BP3 (Supplementary Fig. S5E) indicated a poor prognosis of ICC patients, and the combination of elevated ANLN alongside VIRMA or IGF2BP3 was significantly associated with a deteriorating disease-free survival (DFS) in those ICC patients (Fig. 3K). These results indicated the important roles of these three genes in ICC development.

### ANLN overexpression contributes to ICC tumor growth in vivo and in vitro

**Fig. 4:**
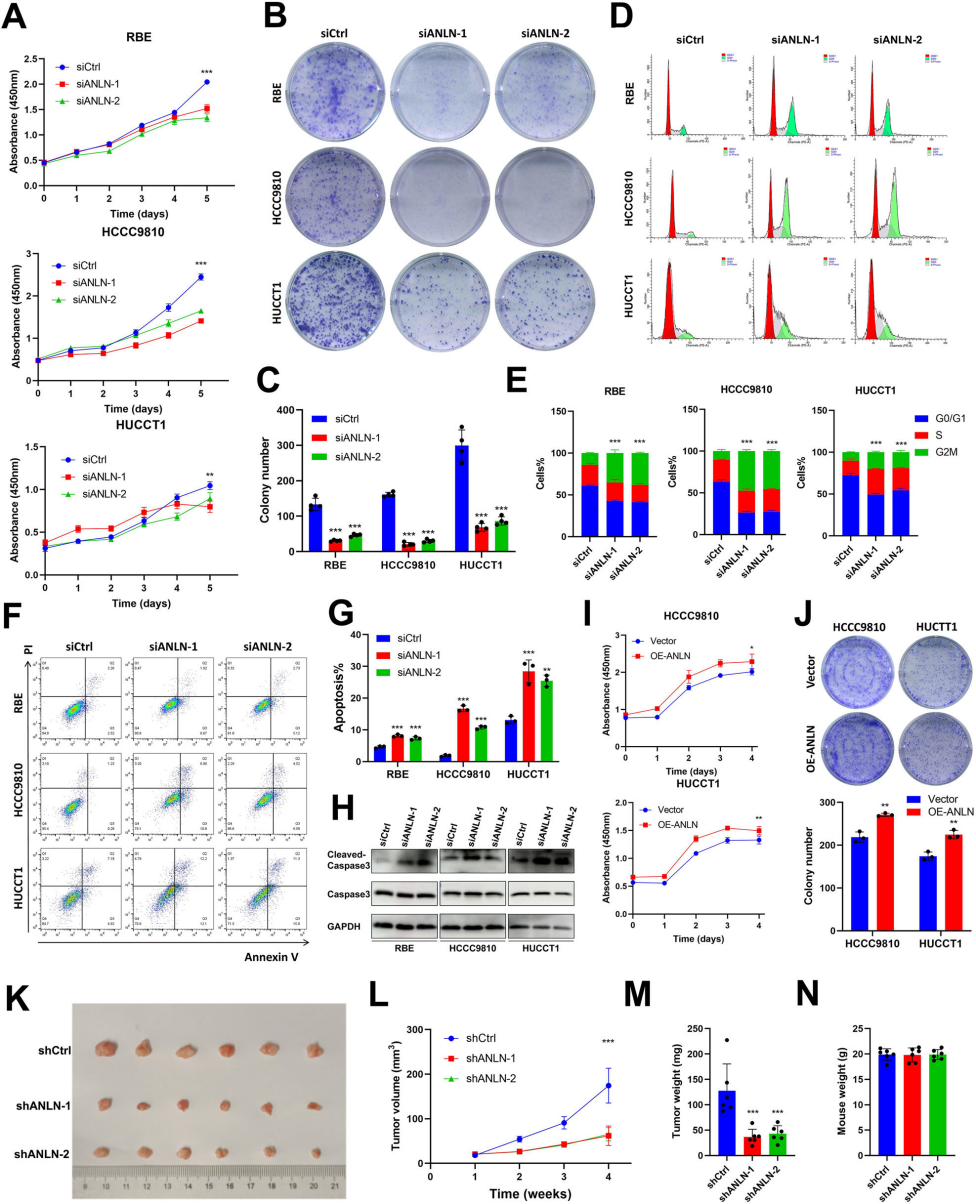
ANLN promotes ICC growth in vitro and in vivo. **A** The cck8 growth curves and **B**, **C** colony formation assay in ICC cells transfected with siANLN or siCtrl. **D**, **E** The effect of ANLN on cell cycle was evaluated by flow cytometry. **F**, **G** Analysis of apoptosis in ICC cells transfected with siANLN or siCtrl by flow cytometry. **H** The expression of Caspase3 in ICC cells transfected with siANLN or siCtrl. **I** The cck8 growth curves and **J**. colony formation assay in vector and ANLN-overexpressing ICC cells. **K** The development of subcutaneous xenografts in HUCCT1 cells transfected with either shNC or shANLN was assessed by measuring (**L)** tumor volume (*n* = 6), **M** tumor weight (*n* = 6), and **N** the weight of the mice (*n* = 6). These results are presented as mean values with standard deviation (SD). *n* = 3-6. *p < 0.05, **p < 0.01, ***p < 0.001.

To clarify the significant role of ANLN in ICC, we silenced or overexpressed ANLN in ICC cell lines using siRNA or plasmids, respectively. The effectiveness was corroborated through qPCR and western blotting analysis (Supplementary Fig. S6A, B). Both cell proliferation and anchorage-dependent colony formation assays revealed that ANLN knockdown decreased ICC cell proliferation in vitro (Fig. 4A–C). ANLN knockdown induced the G2/M phase arrest (Fig. 4D, E) and markedly increased cell apoptosis and cleaved caspase-3 level compared to the negative control (Fig. 4F–H). On the contrary, ANLN overexpression significantly promoted the proliferation and colony formation of ICC cells (Fig. 4I, J). In order to investigate the biological function of ANLN in vivo, we developed a HUCCT1 cell line featuring stable knockdown of ANLN by Lenti-shANLN, which was confirmed by qPCR and immunoblotting (Supplementary Fig. S6C). Examination of subcutaneous xenograft models indicated that ANLN knockdown slowed tumor growth in vivo (Fig. 4K–N). These findings illustrated that ANLN is crucial for ICC cell growth.

### ANLN knockdown leads to mitotic catastrophe by inducing cytokinesis failure and DNA damage in ICC cells

**Fig. 5:**
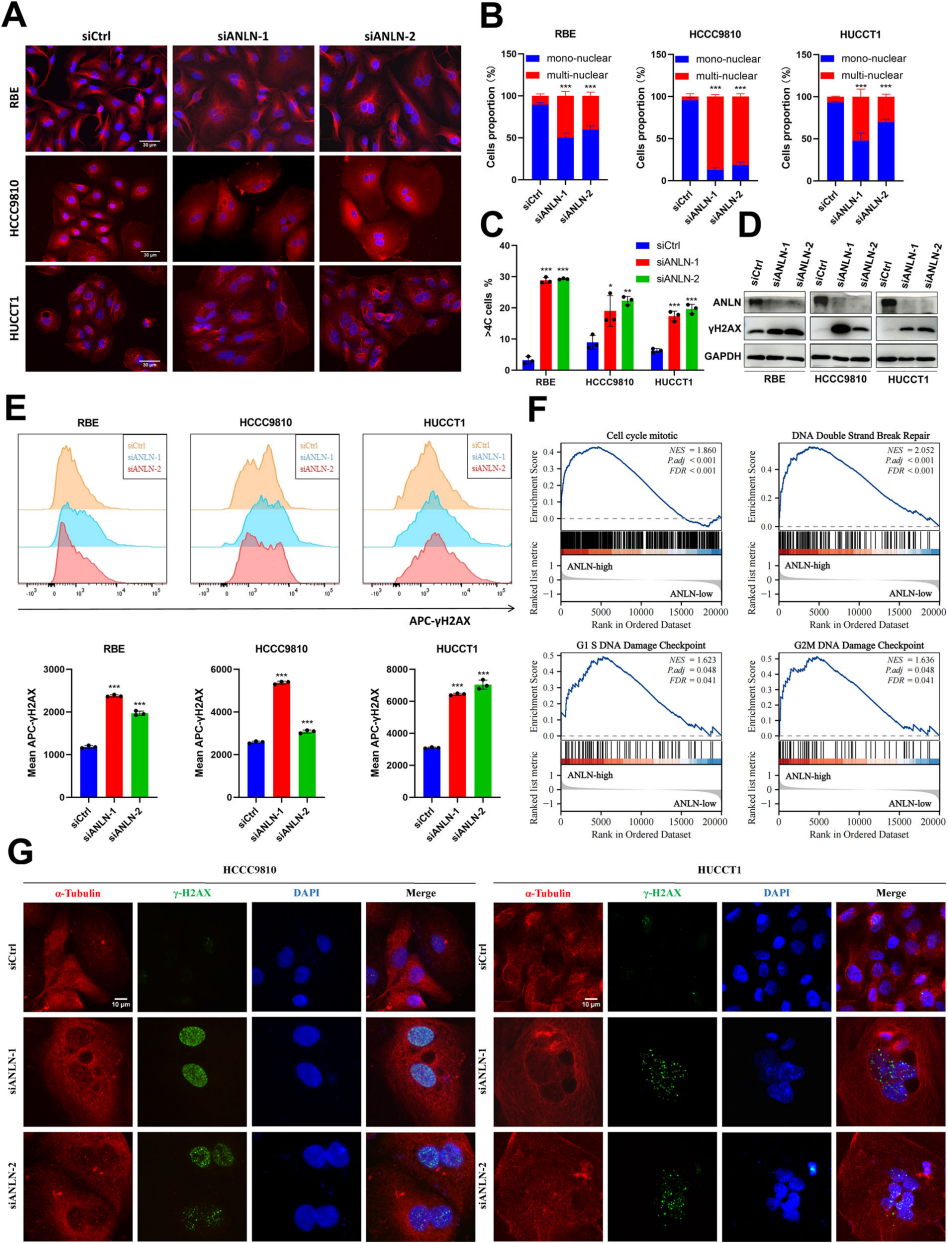
Knockdown of ANLN in ICC cells induced cytokinesis failure and DNA damage. **A** Representative images from immunofluorescence staining displaying α-Tubulin (in red) and DAPI (in blue) within ICC cells that were transfected with either siANLN or siCtrl. Scale bar=30 μm. **B** The histogram illustrating the percentage of mono-nucleated versus multi-nucleated cells. **C** The multiploid proportion of ICC cells transfected with siANLN and siCtrl by flow cytometry. **D** Western blot and **E** flow cytometry measuring the expression of DNA damage marker γH2AX in ICC cells transfected with siANLN and siCtrl. **F** GSEA analysis of the E-MTAB-6389 data in ANLN high-expression group versus ANLN low-expression group. **G** Representative immunofluorescence images of γH2AX foci (green), α-Tubulin (red) and DAPI (blue) in HCCC9810 and HUCCT1 cells transfected with siANLN and siCtrl. Scale bar = 10 μm. These results are presented as mean values with standard deviation (SD). *n* = 3-4. *p < 0.05, **p < 0.01, ***p < 0.001.

Considering the crucial role of ANLN in cytokinesis via RhoA signaling, we aimed to investigate whether ANLN participates in cytokinesis in ICC cells. As previously reported, ANLN expression and sublocalization varied according to the cell cycle, suggesting its potential function in cytokinesis (Supplementary Fig. S7A) [29, 30]. Immunofluorescence analysis indicated that ANLN knockdown resulted in the buildup of bi-nucleated and multi-nucleated cells (Fig. 5A, B). Besides, flow cytometry analysis showed that the polyploidy cell rate was augmented in ANLN-deficient ICC cells (Fig. 5C). It has been reported that multi-nucleated cells are accompanied by chromosome instability and DNA damage. Thus, we examined the effect of ANLN deficiency on DNA damage in ICC cells. ANLN knockdown led to increased expression of γH2AX, which was confirmed by western blotting and flow cytometry analysis (Fig. 5D, E). In addition, GSEA analysis demonstrated that high levels of ANLN in ICC indicated the enrichment of biological behaviors, such as cell cycle mitosis, DNA double-strand break repair, G1/S DNA damage checkpoint and G2/M DNA damage checkpoint, which confirmed our above results (Fig. 5F). Moreover, immunofluorescence analysis exhibited the accumulation of γH2AX foci in ANLN knockdown cells (Fig. 5G and Supplementary Fig. S7B). In brief, ANLN is vital for ICC growth by ensuring the completion of cytokinesis. Conversely, ANLN deficiency leads to cytokinesis failure and DNA damage in ICC cells, thus inducing mitotic catastrophe and suppressing the tumor growth.

### ANLN contributes to ICC growth via suppressing Hippo pathway

**Fig. 6:**
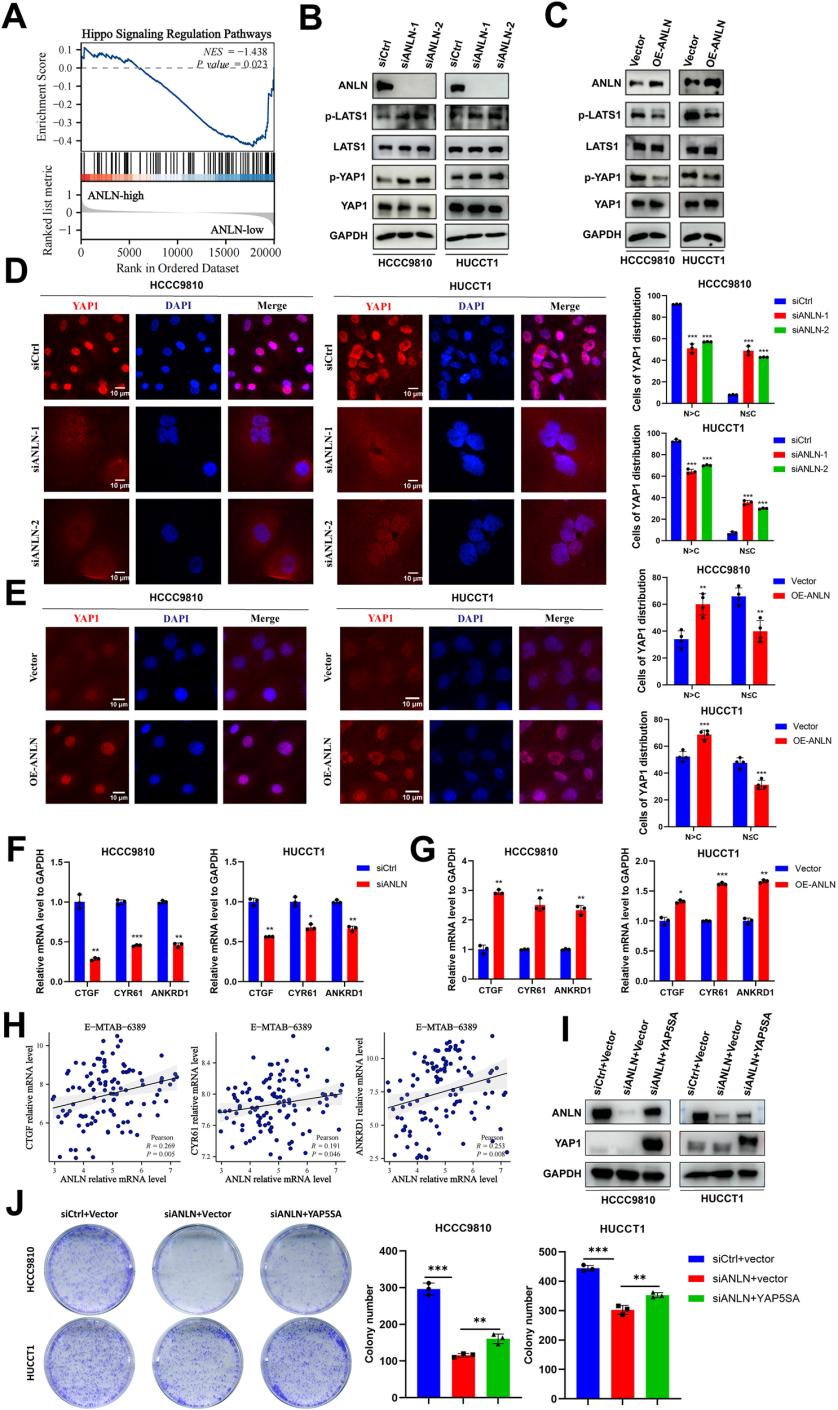
ANLN suppresses the Hippo pathway in ICC cells. **A** Hippo signaling was restrained in ANLN high-expression group based on GSEA analysis from E-MTAB-6389 data. **B** ICC cells transfected with siANLN and siCtrl, **C** The vector and ANLN-overexpressing ICC cells, were subjected to detected the expression of core elements in Hippo signaling (phospho-LATS1 and phospho-YAP1 and total LATS1, total YAP1). **D** Immunofluorescence of YAP1 (red) in siCtrl and siANLN ICC cells treated with enough serum supplement, with YAP1 subcellular localization quantified and showed in the histogram (N nucleus, C cytoplasm). Scale bar=10 μm. **E** Immunofluorescence of YAP1 (red) in vector and ANLN-overexpressing ICC cells treated with serum starvation, with YAP1 subcellular localization proportion exhibited in the histogram. Scale bar = 10 μm. **F** ICC cells transfected with siCtrl and siANLN, **G** Vector and ANLN-overexpressing ICC cells were subjected to determine the mRNA level of YAP1 target genes (CTGF, CYR61, ANKRD1) by qPCR. **H** Pearson correlation between the mRNA levels of ANLN and the mRNA levels of YAP1 target genes (CTGF, CYR61, ANKRD1) in the E-MTAB-6389 dataset. **I** The siCtrl+vector, siANLN+vector and siANLN+YAP5SA ICC cells were subjected to detected the expression of total YAP1 and ANLN. **J** The colony formation assay of siCtrl+vector, siANLN+vector, and siANLN+YAP5SA ICC cells. These results are presented as mean values with standard deviation (SD). *n* = 3–4. *p < 0.05, **p < 0.01, ***p < 0.001.

Next, we attempted to elucidate the additional mechanisms underlying the function of ANLN in ICC growth. Previous studies have reported that cytokinesis failure and RhoA inactivation trigger Hippo signaling activation [5, 23]. Given the essential regulatory role of ANLN in RhoA activation by various studies, thus we hypothesized that ANLN is likely to regulate the Hippo signaling. Here, GSEA analysis revealed the inactivation of Hippo pathway in the high ANLN expression group (Fig. 6A). The Hippo pathway can respond to a series of signals, such as cell-cell contact, mechanical cues, serum, and energy stress, and high cell confluency or serum starvation will lead to the activation of Hippo signaling [5, 31, 32]. With low cell confluency and enough serum supplement, LATS1, the key component of the Hippo pathway, was increased in its phosphorylated form, which augmented YAP1 phosphorylation, suggesting the activation of Hippo pathway in ANLN-deficient ICC cells (Fig. 6B). Oppositely, with high cell confluency, ANLN overexpression restrained the phosphorylation level of LATS1 and YAP1 (Fig. 6C). As illustrated by IF staining, with enough serum supplement, ANLN knockdown inhibited YAP1 cytoplasmic-nuclear translocation in ICC cells (Fig. 6D). Oppositely, under conditions of serum starvation, ANLN overexpression enhanced cytoplasmic-nuclear translocation of YAP1 (Fig. 6E). Next, qPCR results revealed that canonical YAP1 target genes, including CTGF, CYR61, and ANKRD1, were suppressed by ANLN inhibition, and vice versa (Fig. 6F, G). Notably, significant positive correlations between ANLN and YAP1 target genes were observed in the E-MTAB-6389 dataset (Fig. 6H). The above data demonstrate that ANLN activated YAP1 by suppressing the Hippo pathway.

Here, we used the YAP5SA mutant, a constitutively active form of YAP [33, 34], to investigate the interaction relationship between ANLN and YAP1. In ICC cells, ectopical YAP5SA expression markedly increased the mRNA and protein levels of ANLN (Supplementary Fig. S8A, B). Meanwhile, YAP5SA overexpression could rescue the reduced nuclear translocation level of YAP1 mediated by ANLN knockdown (Supplementary Fig. S8C). Surprisingly, YAP5SA overexpression partially enhanced the expression of ANLN in ANLN-deficient ICC cells (Fig. 6I). We further found that YAP1 constitutive activation also could partially rescue the growth inhibition in ANLN-knockdown ICC cell (Fig. 6J). Subsequently, we examined whether phenotypes induced by ANLN overexpression could be abrogated by YAP1 suppression. YAP1 genetic knockdown by siRNA and pharmacological inhibition by CA3 abrogated ANLN-enhanced cell proliferation and colony formation in ICC cells (Supplementary Fig. S9A–D). Collectively, these data indicate that the aberrant ANLN/YAP1 axis enhances ICC cell growth.

### ANLN suppresses Hippo signaling by activating RhoA

**Fig. 7:**
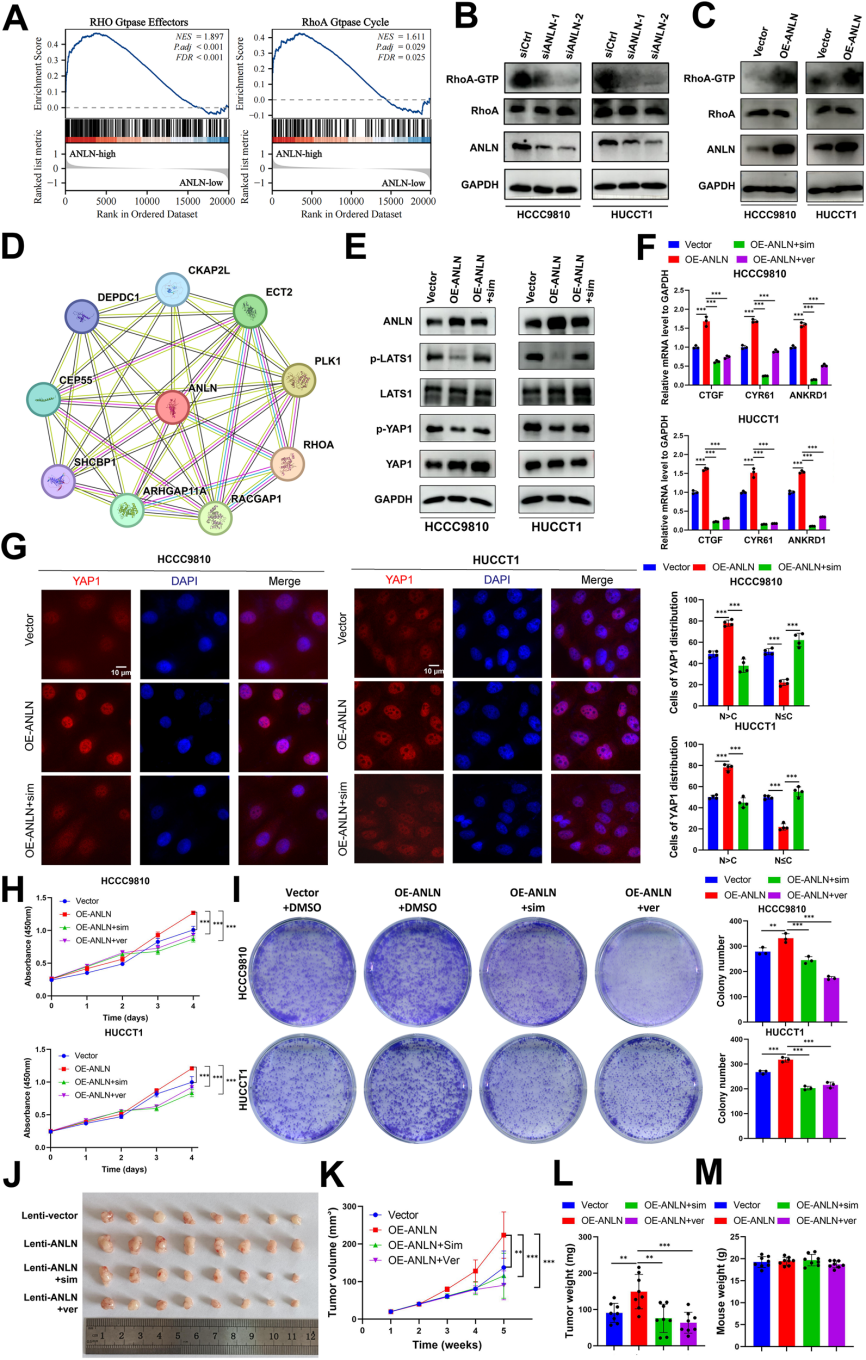
ANLN suppressing Hippo signaling by activating RhoA. **A** RhoA signaling was enriched in ANLN high-expression group based on GSEA analysis from E-MTAB-6389 data. **B** ICC cells transfected with siCtrl and siANLN under conditions of low cell confluency, **C** Vector and ANLN-overexpressing ICC cells under conditions of high cell confluency, underwent a RhoGTPase pull-down assay to assess active RhoA level. **D** PPI analysis among ANLN, RhoA and RhoA regulatory proteins including ECT2, PLK1 and RACGAP1. **E** Western blot was conducted to assess the levels of phospho-LATS1, phospho-YAP1, as well as total LATS1 and YAP1 in vector controls, ANLN-overexpressing cells, and ANLN-overexpressing cells treated with the RhoA inhibitor simvastatin (5 μM). **F** The mRNA expression levels of YAP1 target genes (CTGF, CYR61, and ANKRD1) were analyzed in vector control, ANLN-overexpressing, and ANLN-overexpressing ICC cells treated with the RhoA inhibitor simvastatin (5 μM) or the YAP/TEAD inhibitor verteporfin (10 μM). **G** Immunofluorescence of YAP1 (red) in vector, ANLN-overexpressing ICC cells and ANLN-overexpressing ICC cells treated with simvastatin under conditions of serum starvation, with YAP1 subcellular localization proportion showed in the histogram. Scale bar = 10μm. **H** The cck8 growth curves and **I** colony formation assay in vector, ANLN-overexpressing ICC cells and ANLN-overexpressing ICC cells treated with simvastatin and verteporfin. **J** Growth of subcutaneous xenografts in vector, ANLN-overexpressing ICC cells and ANLN-overexpressing ICC cells treated with simvastatin and verteporfin, with (**K)** tumor volume (*n* = 8), **L** tumor weight (*n* = 8) and **M** mouse weight (*n* = 8) measured. These results are presented as mean values with standard deviation (SD). *n* = 3–8. *p < 0.05, **p < 0.01, ***p < 0.001.

It has been reported that RhoA GTPase inhibits the Hippo signaling pathway and enhances YAP1 transcriptional activity. Meanwhile, RhoA signaling plays an essential role in cytokinesis. Some researchers have illustrated that ANLN acts as the upstream activator of the RhoA pathway through distinct mechanisms. Thus, we speculated that ANLN may regulate the Hippo pathway by activating RhoA signaling. GSEA analysis revealed that biological functions, including RHO GTPase Effectors and RhoA GTPase Cycle, were enriched in the high-ANLN expression group (Fig. 7A). Previous researches illustrated that high cell confluency or serum starvation will lead to the activation of Hippo pathway via suppressing RhoA signaling [31, 32]. Strikingly, ANLN knockdown abrogated activated RhoA levels with low cell confluency, whereas the reverse effect could be caused by ANLN overexpression with high cell confluency (Fig. 7B, C). In addition, the PPI network revealed interactions among ANLN, RhoA, and RhoA regulatory proteins, including ECT2, RACGAP1, and PLK1 (Fig. 7D). Subsequently, we explored whether RhoA is a crucial effector in the regulation of the Hippo pathway by ANLN. As expected, simvastatin, an inhibitor of RhoA GTPase, rescued the levels of p-LATS1 and p-YAP1 that were inhibited by ANLN overexpression under conditions of high cell confluency (Fig. 7E). The mRNA level of canonical YAP1 target genes enhanced by ANLN overexpression could also be reversed by simvastatin and YAP1/TEAD inhibitor verteporfin treatment (Fig. 7F). Moreover, simvastatin treatment resulted in decreased nuclear levels of the YAP1 protein in ANLN overexpressed ICC cells with serum starvation (Fig. 7G). These results confirm our hypothesis.

Subsequently, we assessed the effect of ANLN-induced RhoA activation on the biological characteristics of ICC cells. The RhoA GTPase inhibitors simvastatin or Rhosin and the YAP1 inhibitors verteporfin or CA3 significantly inhibited the proliferation and colony formation abilities of ANLN-overexpressing ICC cells (Fig. 7H, I and Supplementary Fig. S9C, D). Here, we established a stable ANLN-overexpressing cell line (Fig. S10A) to further evaluate the effects of two clinically used drugs including simvastatin and verteporfin on ICC tumors in vivo. The subcutaneous xenograft ICC models demonstrated that simvastatin or verteporfin treatment obviously reduced the tumor volume enhanced by ANLN overexpression, but did not affect the weight of the mice (Fig. 7J–M). Collectively, these results demonstrate that ANLN-mediated RhoA activation is indispensable for Hippo pathway inhibition and malignant phenotypic effects on ICC cells.

### YAP1/TEAD1 transcriptionally activates ANLN to form a positive feedback axis

**Fig. 8:**
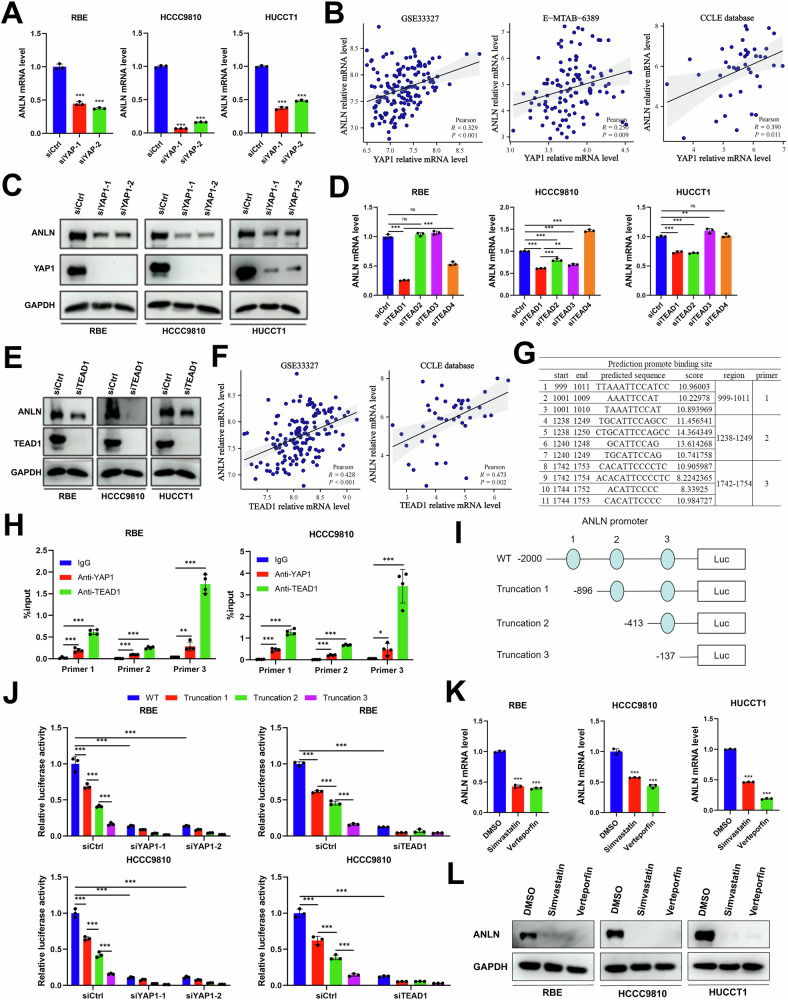
YAP1-TEAD1 transcriptionally activates ANLN to form a positive feedback axis. **A** The mRNA levels of ANLN in RBE, HCCC9810 and HUCCT1 cells transfected with siYAP1 or siCtrl. **B** Pearson correlation analysis of YAP1 mRNA level with ANLN mRNA level in GSE33327, E-MTAB-6389 and CCLE database data. **C** The protein level of ANLN in RBE, HCCC9810 and HUCCT1 cells transfected with siYAP1 or siCtrl by western blot. **D** The mRNA levels of ANLN in RBE, HCCC9810 and HUCCT1 cells transfected with siTEAD1-4 or siCtrl. **E** The protein level of ANLN in RBE, HCCC9810 and HUCCT1 cells transfected with siTEAD1 or siCtrl by western blotting. **F** Pearson correlation of YAP1 mRNA level with ANLN mRNA level in GSE33327 and CCLE database data. **G** The three potential TEAD1-binding regions within the ANLN promoter identified using the JASPAR database. **H** The ChIP-qPCR assay was performed in RBE and HCCC9810 cells using three specific primers along with anti-YAP1, anti-TEAD1, and anti-IgG antibodies. **I** The schematic image for ANLN promoter-reporter plasmids with WT and serial deletion regions. **J** Luciferase activity assays revealed regions in the ANLN promoter that respond to YAP1 and TEAD1. Constructs of the ANLN promoter, which are serially truncated, were transfected into RBE and HCCC9810 cells with either YAP1 or TEAD1 knockdown, as well as control cells, to evaluate relative luciferase activities. **K** The mRNA and **L** protein level of ANLN in ICC cells exposed to DMSO, simvastatin (5 μM) and verteporfin (10 μM). These results are presented as mean values with standard deviation (SD). *n* = 3–4. * *p* < 0.05, **p < 0.01, ***p < 0.001.

Previous studies have suggested that YAP1 regulates the transcriptional expression of cytoskeletal regulators in cancer-associated fibroblasts to promote cancer cell invasion, matrix stiffening and angiogenesis [35]. Thus, we hypothesized that YAP1 probably transcriptionally activates ANLN expression in ICC cells to establish a feed-forward self-reinforcing loop that maintained cellular homeostasis. To test this hypothesis, qPCR was performed. The results revealed that ANLN mRNA levels were decreased in YAP1 knockdown ICC cells (Fig. 8A). In addition, significant positive correlations between YAP1 and ANLN expression were observed in the E-MTAB-6389, GSE33327, and CCLE databases (Fig. 8B). Similar trends were observed for protein levels (Fig. 8C).

Once transported to the nucleus, YAP1 acts as a co-activator to assist the transcription factors TEAD1-4 to promote downstream target gene expression. We aimed to explore the specific transcription factor involved in the regulation of ANLN expression in ICC cells. Conspicuously, TEAD1 knockdown notably reduced ANLN mRNA and protein levels in all three ICC cell lines (Fig. 8D, E). A positive correlation between TEAD1 and ANLN expression was observed in the GSE33327 and CCLE datasets (Fig. 8F). Subsequently, we investigated whether YAP1/TEAD1 regulated ANLN expression via direct transcriptional activation. Based on the prediction of the JASPAR database, the top three potential binding sites for TEAD1 within the ANLN promoter were predicted and specific primers were designed (Fig. 8G). As expected, significant enrichment of YAP1 and TEAD1 in these three putative promoter regions of ANLN was detected by ChIP-qPCR (Fig. 8H). To identify the exact binding sites of TEAD1, promoter-reporter plasmids harboring wild-type and serially deleted regions were constructed (Fig. 8I). Reporter assays indicated that the knockdown of either YAP1 or TEAD1 reduced the luciferase activity associated with the wild-type ANLN promoter, whereas this effect diminished progressively with the truncation promoters (Fig. 8J). Given above results, the ANLN/RhoA/YAP1/TEAD1 positive feedback axis was identified. Thus, we further investigated whether RhoA inhibitors and YAP/TEAD inhibitors regulate the ANLN expression. Apart from the CA3, simvastatin, Rhosin and verteporfin treatment pharmacologically reduced ANLN expression in ICC cells (Fig. 8K, L and Supplementary Fig. S10B, C). Therefore, YAP1-TEAD1 transcriptionally activated ANLN to establish a feed-forward self-reinforcing loop in ICC cells.

## Discussion

Oncogenic transformation and tumor growth requires high-efficiency cell proliferation, which relies on the accurate collaboration of numerous mitosis-related proteins. As a mitosis protein, ANLN recruits contractile ring components to promote the process of cytokinesis, which ensures the M-phase transition. Our study illustrated that ANLN expression was significantly increased in ICC and indicated a poor prognosis. The underlying mechanisms of ANLN overexpression in several tumor types mainly focused on transcriptional and post-translational regulation. For example, the transcription factor OCT1 drives the transcription of ANLN, and so does Wnt/β-catenin-dependent TCF [36, 37] and MYC [38]. In addition, regulation of ANLN protein levels refers to ubiquitin-mediated proteasomal degradation during cell cycle progression [14, 29]. However, the epigenetic regulation of ANLN expression remains unclear.

The epigenetic regulatory mechanism of m6A modification is an emerging research frontier in tumor biology. Recent reports have demonstrated the vital functions of m6A modulators in tumor growth. Researches have examined the m6A writer VIRMA and the reader IGF2BP3 in relation to the progression of several types of cancer, including liver, breast, and lung cancers [26, 39–42]. Here, we discovered that VIRMA is essential for the m6A modification of ANLN mRNA in ICC. Subsequently, IGF2BP3 was confirmed as an important m6A reader that promotes the stability of m6A-modified ANLN mRNA, which accounted for ANLN overexpression in ICC at the post-transcriptional level. Crucially, the combination of ANLN and VIRMA or IGF2BP3 offers a greater predictive value compared than each marker alone, highlighting the enhanced accuracy of these genes as biomarkers in forecasting the prognosis of ICC patients.

Previous researches have mainly focused on the conservative role and underlying mechanism of ANLN in cytokinesis and the cytoskeleton system. Meanwhile, targeted inhibition of ANLN was proved to significantly suppress the malignant phenotypes in vitro and in vivo models of various tumors. Recently, increasing studies have discovered novel effects of ANLN on tumor development. ANLN has been reported to function as an upstream regulator of PI3K/AKT signaling to facilitate disease progression in gallbladder cancer [43]. In addition, ANLN could synergistically promote IGF2BP1-induced the protein stability of the proto-oncogene c-Myc and the activation of the MAPK signaling pathway, which accelerated prostate cancer growth [44]. Likewise, the progression of pancreatic cancer was influenced by ANLN through the miR-218-5p/LASP1 signaling pathway, which is mediated by EZH2 [15]. Startlingly, nuclear ANLN has been shown to form a transcriptional complex with SP1, thus enhancing KIF2C transcriptional activity and activating the mTORC1 pathway, which leads to the hepatocellular carcinoma bone metastasis [45]. Moreover, ANLN promoted doxorubicin resistance through direct interaction with RhoA in breast cancer cells [46]. In head and neck squamous cell carcinoma, ANLN activates the ERK-MAPK pathway, thus upregulating PD-L1 level, which contributes to an immunosuppressive tumor environment [47]. According to a recent study, nuclear ANLN can enhance transcription initiation-related Pol II clustering, and further regulates the expression of various target genes related with oxidoreductase activity and cell differentiation [48]. These studies demonstrated the new and expanded roles of ANLN in tumors progression, yet the biological function and mechanism of ANLN in ICC remains rare.

**Fig. 9:**
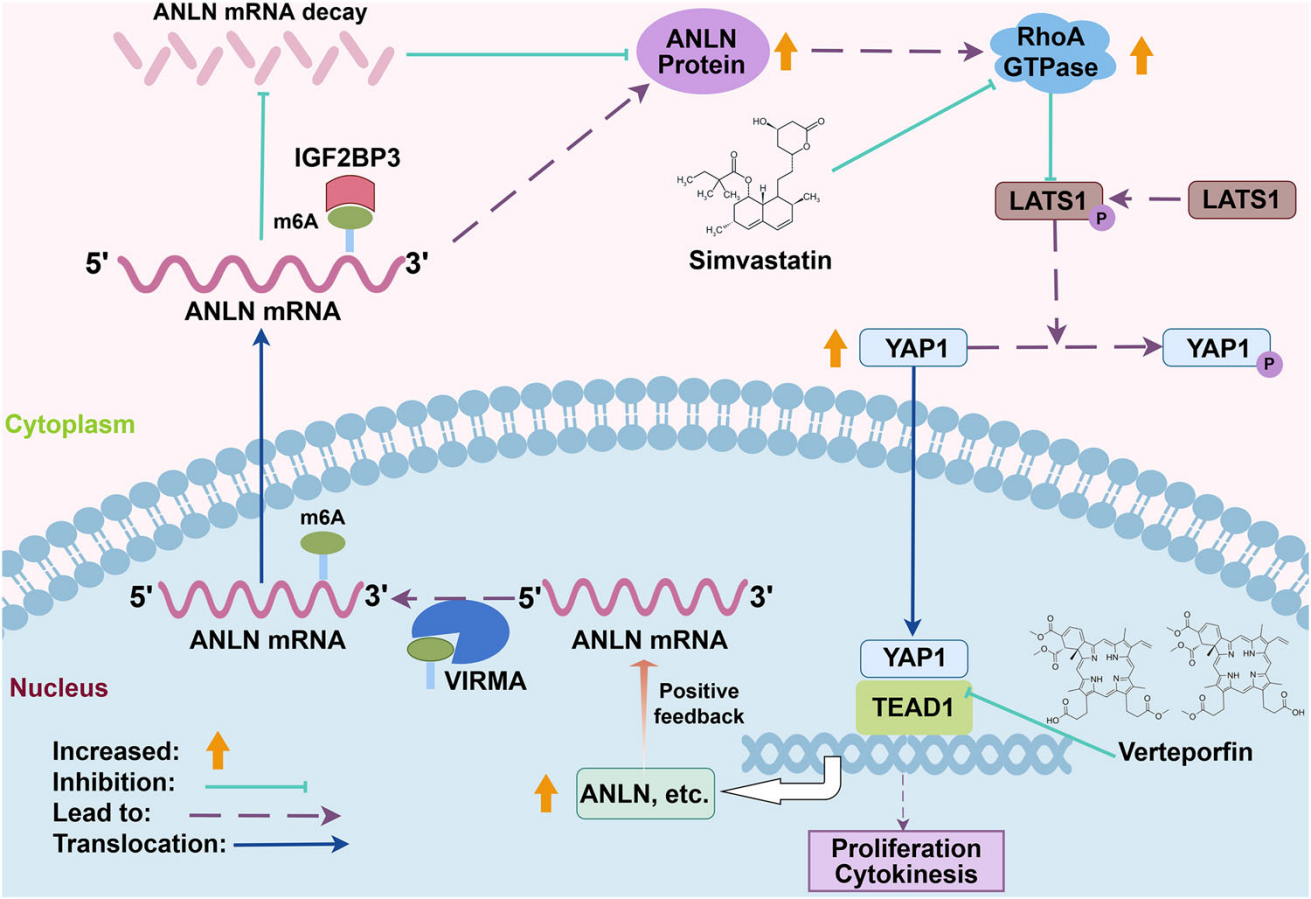
Functional regulation of ANLN in ICC. VIRMA increased the m6A modification mRNA level of ANLN, which was recognized by IGF2BP3, thus promoting mRNA stability. Elevated ANLN enhanced RhoA GTPase activity and subsequently triggered YAP1-TEAD1 transcriptional activity by restraining the phosphorylation level of LATS1 and YAP1. The RhoA/YAP1/TEAD1 axis further activated the transcriptional expression of ANLN to form a feed-forward self-reinforcing loop, thus enhancing cell proliferation and ensuring cytokinesis. The RhoA inhibitor simvastatin and YAP/TEAD inhibitor verteporfin were determined to be the disruptors of this signaling axis, signifying its prospective utility in ICC therapy.

In this study, we illustrated that ANLN promoted ICC growth by ensuring cytokinesis and restraining the Hippo pathway. Moreover, we found that ANLN knockdown leads to cytokinesis failure, cell polyploidy and increasing replication stress, further resulting in intense DNA damage, genomic instability, and cell death known as mitotic catastrophe, which potentially also is one of the essential mechanisms for tumor suppression mediated by ANLN inhibition [49, 50]. Mechanically, ANLN-mediated RhoA activation collectively regulates cytokinesis and the Hippo pathway. In addition, ANLN was identified as the target gene that is transcriptionally regulated by YAP1-TEAD1, thus forming a feed-forward loop. This loop augmented RhoA/YAP1/TEAD1 signaling activity to facilitate the proliferative properties of ICC cells and maintain ANLN overexpression, which safeguarded the natural accomplishment of cytokinesis (Fig. 9).

Cytokinesis, the final step of the cell cycle, ensures mitosis fidelity, which is of great significance for sustaining cell proliferation and tumor growth. Regulated by RhoGEFs and RhoGAPs, active RhoA is required for cytokinesis [10]. In this study, we demonstrated that ANLN serves as the upstream activator of RhoA signaling, which is consistent with previous biological and oncological research. As a GTPase protein, RACGAP1 is demonstrated to enhance RhoA activation by direct interaction with ECT2, a RhoGEF contributing to RhoA activation [51]. Meanwhile, PLK1 is responsible for RACGAP1 phosphorylation that is crucial for RhoA activation [52]. ANLN has been reported to work as a scaffold protein to ensure the physical interaction between RACGAP1 and PLK1, thus promoting PLK1-mediated RACGAP1 phosphorylation and RhoA activation [12]. Furthermore, ANLN can interact with RhoA activator ECT2 to form a complex that stabilizes central spindle microtubules and maintains the activity of RhoA [8, 11]. In addition to binding to RhoA regulatory proteins, ANLN also directly interacts with RhoA through its anillin homology domain, which is crucial for stabilizing RhoA activation and accurate location [13, 24, 46, 53, 54]. The above various reasons potentially account for the mechanism underlying ANLN-mediated RhoA activation.

As the core element of the Hippo pathway, YAP1 is vital for driving oncogenic activities, including cellular growth and metastasis. As is well known, the Hippo pathway can be regulated by multiple biological stimulations incorporating cell-cell interaction, lysophosphatidic acid, serum, and mechanical cues [18, 19]. RhoA is a crucial regulatory element in the Hippo pathway in response to upstream signals [55]. Here, we illustrated that ANLN restrains the Hippo pathway by reducing YAP1 phosphorylation level and enhancing YAP1 transcriptional activity, partly explaining the promoting-tumor effects of ANLN in ICC. Then we further identified that ANLN-mediated Hippo signaling suppression is dependent on active RhoA-GTP, which is also crucial for ANLN-mediated promoting-tumor in ICC. In this study, we demonstrated that ANLN contributed to ICC growth via the RhoA/LATS1/YAP1 axis, establishing a link between ANLN and the Hippo pathway, which broadened the understanding of the role of ANLN in tumor.

YAP1, the core element of the Hippo pathway, functions as a transcription co-activator that interacts with transcription factors TEAD1-4 to activate downstream target genes. In this study, we used both genetic and pharmacological approaches to confirm that YAP1 and TEAD1 cooperatively promoted ANLN expression at transcriptional level in ICC, thus establishing a feed-forward loop between ANLN and Hippo pathway via RhoA signaling, which deepened the significance of our research. Obviously, this positive feedback axis was of importance for ICC growth. Furthermore, it also gave us some insight into the relationship between the Hippo pathway and cytokinesis. Previous reports have illustrated the role of YAP1 in cytokinesis via transcriptional regulation of TPR and physical co-localization with the polarity protein PATJ [5, 56]. Moreover, a recent study demonstrated that YAP1 collaborates with the mitotic spindle and midbody to ensure mitotic division [57]. Thus, the Hippo pathway may be closely related to the process of cytokinesis, and further deeper research is deserved. Here, our findings identified the ANLN/RhoA/YAP1/TEAD1 positive feedback axis in ICC, which was essential for cytokinesis and tumor growth.

Simvastatin is a lipid-lowering agent clinically applied in cardiovascular disease, which blocks the transformation of HMG-CoA to mevalonate by competitively suppressing HMG-CoA reductase and reducing cholesterol synthesis [58]. Furthermore, simvastatin is reported to inhibit tumor growth by repressing the activation of RhoA GTPase and nuclear translocation of YAP1 [59, 60]. Meanwhile, Verteporfin is a photosensitizer employed in photodynamic therapy to eliminate abnormal blood vessels in ophthalmic diseases, including age-related macular degeneration and pathologic myopia [61]. Verteporfin is determined as a YAP1 inhibitor that disrupts the YAP1-TEAD interaction, thus abrogating their transcriptional activation capacity. Moreover, the pharmacological inhibition of YAP1 mediated by verteporfin can reduce progression and overcome drug resistance in various tumors [62, 63]. Considering both YAP inhibitor verteporfin and RhoA inhibitor simvastatin are clinical drugs, both two inhibitors were used in the functional rescue experiments both in vitro and in vivo, although they are not primarily exploited for anticancer therapy in clinical work, just has been documented by several preclinical studies. Nevertheless, the present evidence supporting their curative effect and potential mechanism for ICC is limited.

Currently, there is no specific molecular inhibitor directly targeting ANLN. Alternatively, exploring possible medications targeting crucial elements of the signaling axis could serve as a promising therapeutic strategy for cancer treatment. Here, in addition to inhibiting active RhoA and YAP1 transcriptional activity known in previous studies, simvastatin or verteporfin treatment also further reduced the mRNA and protein levels of ANLN in ICC, subsequently destroying the stability of the ANLN/RhoA/YAP1/TEAD1 feed-forward loop. Of note, both simvastatin and verteporfin reversed the proliferative characteristics of ANLN-overexpressing ICC cells in vivo and in vitro. These results indicated promising therapeutic effects for simvastatin and verteporfin on ICC (Fig. 9). Moreover, simvastatin and verteporfin have been reported in significant efficacy to overcome tyrosine kinase inhibitors resistance mediated by YAP1 signaling [16, 64]. Additionally, recent studies have revealed their potential effects on altering tumor environment in favor of anti-tumor immune responses [16, 65, 66]. Considering above studies, simvastatin and verteporfin have great prospects for clinical tumor treatment, and future studies on combination drug therapy or immunotherapy are expected in ICC.

In summary, our findings illustrated that VIRMA/IGF2BP3-mediated m6A modification regulates ANLN expression in ICC. Furthermore, elevated ANLN promoted cell proliferation and cytokinesis dependent on the RhoA/YAP1 axis. In addition, ANLN expression can also be reinforced by the RhoA/YAP1/TEAD1 feed-forward loop, indicating a promising strategy for ICC treatment by targeting this signaling axis (Fig. 9).

## Materials and methods

### Integrated bioinformatic analyses

The RNA-sequencing data were obtained from The Cancer Genome Atlas (TCGA) database, Gene Expression Omnibus (GEO) database (GSE32879, GSE33327, GSE26566, GSE76297), and E-MTAB-6389 datasets. A total of 199 mitotic spindle-related genes were obtained from the Molecular Signatures Database. Protein (ANLN, VIRMA, IGF2BP3) expression data were downloaded from the cProSite Cancer Proteogenomic database. The cBioPortal genomics database was applied to analyze the DNA amplification or mutations of ANLN. The SRAMP database was used to predict potential m6A sites. The JASPAR database was employed to predict the transcriptional regulatory sites. The CCLE database was used to acquire the expression profile of cholangiocarcinoma cell lines.

The DESeq2 program or limma algorithm was used for the differential analyses with an adjusted p value less than 0.05 as the standard. The mRNA or protein expression correlation was determined by Pearson correlation analysis. A comparison using Gene Set Enrichment Analysis (GSEA) was used to compare the high-ANLN and low-ANLN expression groups. The STRING database was employed for the construction of the protein-protein interaction (PPI) network.

### Patients and clinical samples

A total of 25 matched pairs of ICC tumors and corresponding adjacent normal tissues were obtained from the Cancer Hospital, Chinese Academy of Medical Sciences, for mRNA and protein extraction to measure the level of ANLN. Immunohistochemical staining was performed on paraffin-embedded sections from a group of 98 ICC patients. Overall survival (OS) was defined as the period from surgery to death, whereas relapse-free survival (RFS) was defined as the time from surgery to cancer recurrence. Patients who were alive without relapse were recorded as censored on the date of their last follow-up or death, and the clinicopathological characteristics of the ICC cohort were acquired. Meanwhile, the clinicopathological characteristics of this ICC cohort were acquired.

The research was conducted in accordance with the principles of the Declaration of Helsinki and was approved by the Institutional Review Board of the Cancer Hospital at the Chinese Academy of Medical Sciences. All participating patients provided informed consent prior to their involvement in the study.

### Cell lines and cell culture

Human ICC cell lines, namely RBE, HCCC9810, and HUCCT1, were maintained in our laboratory and their identities were confirmed through short tandem repeat (STR) analysis. The RBE and HCCC9810 cell lines were propagated in Roswell Park Memorial Institute (RPMI) 1640 medium, whereas HUCCT1 cells were cultured in Dulbecco’s Modified Eagle Medium (DMEM). Both media were supplemented with 10% fetal bovine serum, 100 U/mL streptomycin, and 100 U/mL penicillin. These cultures were incubated at 37° C in a 5% CO_2_ atmosphere.

### siRNA and plasmid transfection

Gene-specific siRNAs (siANLN, siVIRMA, siIGF2BP1-3, siYAP1, and siTEAD1-4) and a negative control siRNA (siCtrl) were purchased from GenePharma (Suzhou, China). The specified ICC cells were transfected with Lipofectamine RNAiMAX (Invitrogen, Carlsbad, CA, USA) and siRNA mixture following the manufacturer’s instructions. A complete list of siRNA sequences is provided in Supplementary Table S2.

The empty vector (pCMV) and gene-specific plasmids (pEnCMV-ANLN) were obtained from Miaoling (Wuhan, China). The GPL4-ANLN promoter plasmid (ANLN-WT) and the corresponding truncation plasmids (ANLN-truncation 1-3) were acquired from GenePharma (Suzhou, China). The ICC cells were transfected with the vector by Lipofectamine 3000 (Invitrogen, Carlsbad, CA, USA) following the manufacturer’s instructions.

### Quantitative real-time PCR (qPCR) assay

Total RNA was extracted from ICC cells using the RNA-Quick Purification Kit (Yishan Biotechnology, Beijing, China), according to the manufacturer’s protocol. A Nanodrop 2000 spectrophotometer (Thermo Fisher, USA) was used for the measurement of concentration and quality. For cDNA synthesis, PrimeScript RT Master Mix (Takara, Japan) was employed to reverse transcribe 0.5–2 µg of total mRNA, which was then amplified through RT-qPCR using Power SYBR Green PCR Master Mix (Applied Biosystems, USA). The relative expression levels of the target genes were determined using the 2^−ΔΔCt^ method and normalized to the reference genes. Primer sequences are listed in Supplementary Table S3.

### Western blotting analysis

In short, the tumor tissues and cells were lysed using RIPA lysis buffer (APPLYGEN, Beijing, China) containing 2% protease and phosphatase inhibitors (MedChemExpress) and kept on ice. The BCA assay kit (APPLYGEN, Beijing, China) was utilized to determine the protein concentration. Protein extracts were separated through sodium dodecyl sulfate polyacrylamide gel electrophoresis (SDS-PAGE), followed by transferring onto a nitrocellulose membrane. Subsequently, the nitrocellulose membranes underwent incubation with specific primary antibodies and then secondary antibodies. A detailed list of the antibodies is provided in Supplementary Table S4.

### Dual-luciferase reporter assay

For the identification of transcription factors, the DNA sequences containing the promoter region of ANLN (−2000 to +100) were cloned into the GPL4-luc plasmid (marked as ANLN-WT). The potential TEAD1 binding sites within the ANLN promoter were further truncated in GPL4-ANLN-truncation-luc (designated as Truncation 1-3). The ICC cells were co-transfected with pRL-TK-Renilla-luc plasmids and ANLN-WT or Truncation 1-3. Subsequently, a dual-luciferase reporter assay was conducted using the Dual-Luciferase Reporter Assay System (Promega, Wisconsin, USA) following the manufacturer’s instructions.

### Cell proliferation assay

Cell viability was determined utilizing a Cell Counting Kit-8 (CCK8) assay (Biosharp Life Science, China). ICC cells were plated at a concentration of 3 × 10^3^ cells/well in 96-well plates. Simvastatin (YAP1-TEAD inhibitor, 5 μM), verteporfin (RhoA inhibitor, 10 μM), CA3 (YAP1-TEAD inhibitor, 1 μM), and Rhosin hydrochloride (RhoA inhibitor, 50 μM) were procured from MedChemExpress. Following each treatment, the CCK-8 solution was sequentially introduced into each well and incubated for 1 h with ICC cells. Absorbance was then measured at 450 nm. In the colony formation test, ICC cells from each experimental group were seeded at a density of 3 × 10^3^ cells/well in 6-well dishes and incubated for 7-10 days. Cells were dyed with a mixture 4% paraformaldehyde and crystal violet (Solarbio, Beijing, China).

### Flow cytometry

The rate of cell apoptosis was measured by employing an Annexin V-APC/PI kit (MULTISCIENCES, China) as per the protocol provided by the manufacturer. We applied Flow Jo V10 software for the data processing. The APC^+^/PI^+^ and APC^+^/PI^-^ cells were considered apoptotic cells. To analyze the cell cycle, ICC cells were assessed using flow cytometry to establish the distribution of the distinct phases of the cell cycle. The Cell Cycle Staining Kit (MULTISCIENCES, China) was utilized according to the provided guidelines. To detect the DNA damage level, ICC cells were collected and incubated with γH2AX antibody (Biolegend, 613415) for 1 h. The Flow Jo V10 software was applied for the data processing.

### Lentivirus transfection

Lentiviral vectors, including empty vector (pLV3-CMV-MCS) and gene-specific plasmids (pLV3-CMV-ANLN and pLV3-CMV-YAP5SA) for overexpression, were obtained from Miaoling (Wuhan, China). The lentiviral vectors for knockdown (lenti-shCtrl, lenti-shANLN-1, and lenti-shANLN-2) were obtained from GenePharma (Suzhou, China). Briefly, 293 T cells were used for the acquisition of lentiviruses.

### Methylated RNA immunoprecipitation (MeRIP)

The MeRIP experiments were conducted in ICC cells according to the guidelines provided by the manufacturer (BiosinBio MeRIP m6A Kit). To begin, the RNA extracted from ICC cells was fragmented into 300 nt fragments using the Fragmentation buffer. Total RNA was divided into an input group and an IP (IgG or m6A) group at a ratio of one to eight. Subsequently, the fragmented RNA was incubated with an anti-m6A or IgG antibody for 4 h at 4 °C with rotation. Following this, the RNA bound to the anti-m6A antibody was combined with washed magnetic beads and collectively incubated for 1 h at 4 °C with rotation. The complexes were then washed three times, and the RNA that was bound to the beads was extracted using a mixture of phenol, chloroform, and isoamyl alcohol (25:24:1). The Eluted RNA was then subjected to qPCR analysis.

### RNA immunoprecipitation (RIP) assay

RIP assays were conducted as per the guidelines provided by the manufacturer (BiosinBio RIP Kit). Briefly, cell lysis was treated using the DNase salt stock for DNA removal. Following this, the cell lysates were pre-coated and incubated with 5 mg of anti-IGF2BP3 antibody (Proteintech) or IgG at 4 °C with rotation overnight. Then, the IP antibody-bound RNA was then added to the washed magnetic beads. The immunoprecipitated RNA-protein complex was purified using TRIzol for RNA extraction, which was subjected to qPCR.

### Immunofluorescence (IF)

ICC cells were fixed in 4% formalin at room temperature for a duration of 10 min. The cells underwent a 10-minute incubation with 0.5% Triton X-100. After blocking with goat serum for 1 h, ICC cells were exposed to primary antibodies (ANLN, VIRMA, IGF2BP3, α-tubulin, γH2AX, and YAP1) overnight at 4 °C. Subsequently, the ICC cells were incubated with either goat anti-mouse IgG Alexa Fluor 488 or goat anti-rabbit IgG Alexa Fluor 594 (Proteintech) for 1 h at room temperature. The final step involved staining the ICC cells with 4,6-diamidino-2-phenylindole (DAPI), and visualization was performed by using a fluorescence microscope.

### Chromatin immunoprecipitation (ChIP) assay

The ChIP assay was performed in RBE and HCCC9810 cells by applying a ChIP qPCR Kit (Cell Signaling Technology). Briefly, ICC cells were fixed in 37% formaldehyde for cross-linking. The lysate was treated with Micrococcal Nuclease to digest DNA, and then sonicated for 3 sets of 20-sec pulses to break the nuclear membrane. Immunoprecipitation of the supernatants was then carried out using the Histone H3 antibody as a positive control and IgG as a negative control, along with YAP1 and TEAD1. The antibody-bound DNA was then mixed with Protein G magnetic beads. The bound DNA was washed and purified. Last, the eluted DNA was subjected to qPCR.

### RhoGTPase pull-down assay

The RhoA pull-down activation assay biochem kit (Cytoskeleton Inc., USA) was utilized for the performance of Rho GTPase pull-down assays following the manufacturer’s instructions. RhoA activation was assessed by performing a pull-down assay using GST-RBD beads, followed by detection using western blotting analysis.

### Immunohistochemistry (IHC)

To summarize, tissue samples embedded in paraffin were treated to remove paraffin and moisture. Following antigen repair and blockage, the samples underwent overnight incubation at 4 °C with primary antibodies (ANLN, VRIMA, IGF2BP3). Next, the samples were incubated with a secondary antibody conjugated with horseradish peroxidase (HRP) for 1 h at 37 °C, followed by staining with diaminobenzidine (DAB). Semi-quantitative analysis of the IHC sections included assessment of both the proportion of positive cells and the intensity of positive staining. The scoring of all sections followed the methodology outlined in previous study [67].

### Tumor xenograft assay

Five-week-old female BALB/c nude mice were acquired from the Vital River Laboratory Animal Technology Co., Ltd. Transfected HUCCT1 cells were subcutaneously injected into the axilla of mice (100 μL containing 5 × 10^6^ cells in PBS). Tumor volumes were measured per week (0.5× length × width^2^). At the end of either 4 or 5 weeks, all mice were sacrificed, and the tumors were excised for weighing.

To investigate the impact of simvastatin and verteporfin treatments on ICC growth in vivo, xenograft mouse models were randomized and treated with either simvastatin (5 mg/kg body weight, administered via intraperitoneal injection, five times a week) or verteporfin (100 mg/kg body weight, administered intraperitoneal injection every two days) individually [59, 60]. All animal-related procedures adhered to the internal biosafety and bioethics guidelines of the Institutional Animal Care and Use Committee (IACUC) of the National Cancer Center/Cancer Hospital.

### Statistical analysis

Statistical analyses were conducted utilizing the Prism GraphPad software (GraphPad Software Inc., San Diego, CA, USA). The results are presented as mean values with standard deviation (SD). To analyze overall survival, the Kaplan–Meier method was employed in conjunction with Cox regression analysis. To explore the relationship between ANLN level and clinicopathological characteristics, the package “ stats” was applied to the baseline data sheet. The package “survival” was used for univariate analysis and multivariate analysis. Differences between the control and experimental groups were assessed using either Student’s *t*-test or ANOVA, as appropriate, with significance levels indicated as *P < 0.05, **P < 0.01, and ***P < 0.001. The numbers in bars indicate the number of cases in each category.

## Supplementary information

Supplementary Figs. S1–10

Supplementary Tables

Original western blot images

## Data Availability

All data are available upon request. All data generated or analyzed during this study are included in this article and its supplementary material files. Further inquiries can be directed to the corresponding authors.
