# Supplementary material for: VIRMA/IGF2BP3-mediated ANLN upregulation promotes intrahepatic cholangiocarcinoma growth by forming a positive feedback loop with RhoA/YAP1/TEAD1 signaling pathway
Source: Cell Death Dis. 2026 Jan 9;17(1):20. doi: 10.1038/s41419-025-08197-5 (PMC12789643; doi:10.1038/s41419-025-08197-5)
Supplement: Supplementary file 1 — Supplementary Figs. S1–10 [file 41419_2025_8197_MOESM1_ESM.docx]

**VIRMA/IGF2BP3-mediated ANLN upregulation promotes intrahepatic** **cholangiocarcinoma growth by forming a positive feedback loop with RhoA/YAP1/TEAD1 signaling pathway**

Jiajun Zhang^1^, Ning Huang^1^, Lin-rui Gao^2^, Ai Guo^2^, Hongming Deng^2^, Liming Wang^1,^*****, Mei Liu^2,^*****

^1^Department of Hepatobiliary Surgery, National Cancer Center/National Clinical Research Center for Cancer/Cancer Hospital, Chinese Academy of Medical Sciences and Peking Union Medical College,Beijing, China

^2^Laboratory of Cell and Molecular Biology & State Key Laboratory of Molecular Oncology, National Cancer Center/National Clinical Research Center for Cancer/Cancer Hospital, Chinese Academy of Medical Sciences and Peking Union Medical College, Beijing, China

*** Correspondence should be addressed to:**

Liming Wang

Department of Hepatobiliary Surgery, National Cancer Center/National Clinical Research Center for Cancer/Cancer Hospital, Chinese Academy of Medical Sciences and Peking Union Medical College, 17 Panjiayuan Nanli, Chaoyang District, Beijing 100021, China

Mobile phone: +86-13161361180; Email: [stewen_wang@sina.com](mailto:stewen_wang@sina.com)

Mei Liu, Ph.D

Laboratory of Cell and Molecular Biology & State Key Laboratory of Molecular Oncology, National Cancer Center/National Clinical Research Center for Cancer/Cancer Hospital, Chinese Academy of Medical Sciences and Peking Union Medical College, 17 Panjiayuan Nanli, Chaoyang District, Beijing 100021, China

Tel: +8610 87788487; Fax: +8610 67738220;

E-mail: [liumei@cicams.ac.cn](mailto:liumei@cicams.ac.cn)

**Supplementary Figures and Figure Legends**

**
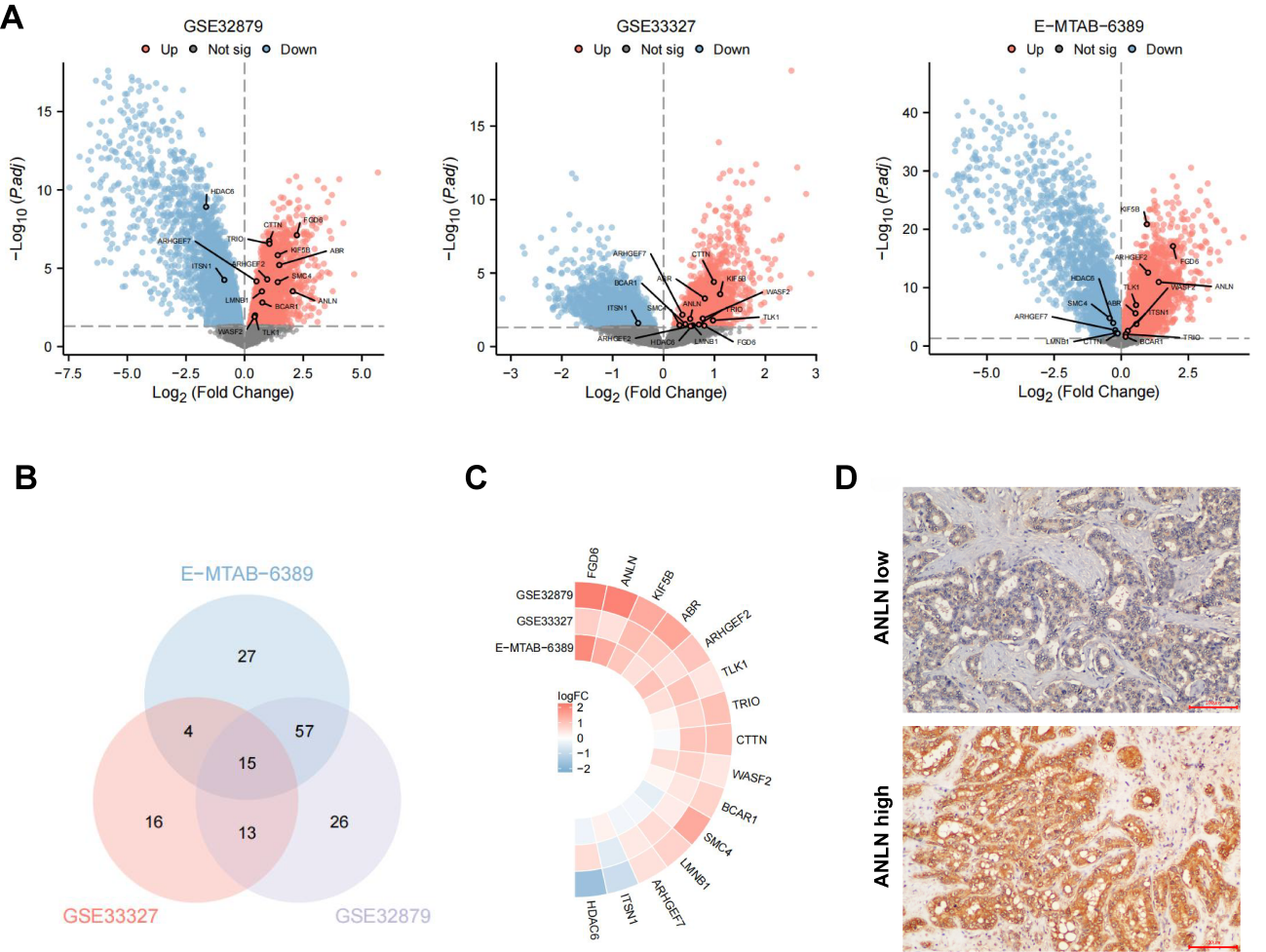
**

**Figure S1.**

1. Differentially expressed genes (DEGs) were identified in E-MTAB-6389, GSE32879, GSE33327 ICC data sets. **B.** Venn diagram of DEGs collectively enriched in screening analysis according to three ICC data sets. **C.** The expression profiles of the top 15 mutual DEGs associated with three ICC datasets are analyzed. The mRNA level changes of these genes are determined using log2 transformation, comparing tumor tissues with adjacent non-tumor tissues. **D**. Representative images showing staining of ANLN of low group and high group in samples from ICC patients. Scale bar = 100μm.


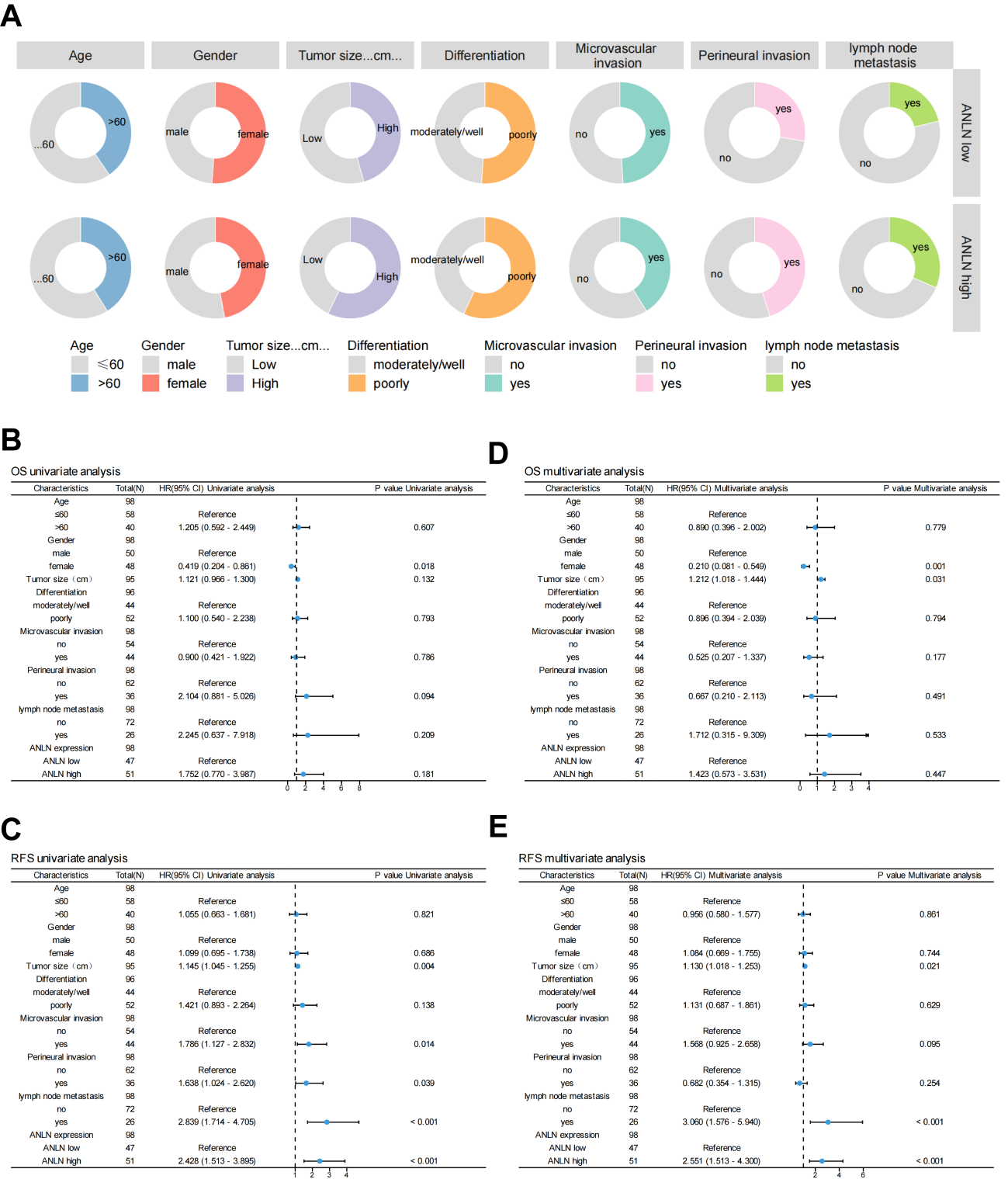


**Figure S2**

**A.** Clinicopathological characteristics of ICC cohort between ANLN high group and ANLN low group. **B.** Univariate analysis of clinicopathological characteristics and ANLN expression level for OS in ICC cohort. **C.** Univariate analysis of clinicopathological characteristics and ANLN expression level for RFS in ICC cohort. **D.** Multivariate analysis of clinicopathological characteristics and ANLN expression level for OS in ICC cohort. **E.** Multivariate analysis of clinicopathological characteristics and ANLN expression level for RFS in ICC cohort.

**
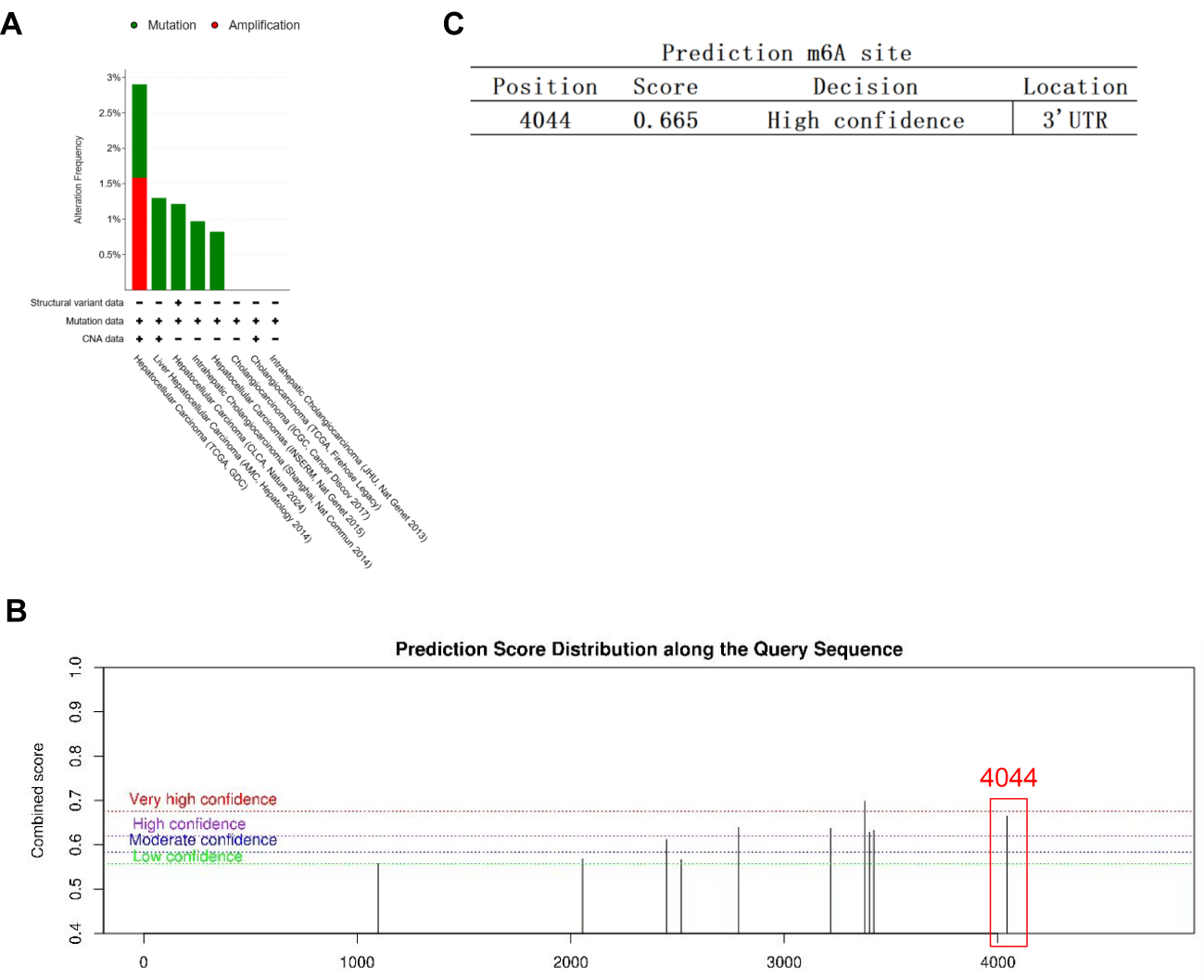
**

**Figure S3**

**A.** Analysis of genetic alterations of ANLN in hepatobiliary malignancies using cBioPortal genomics database. **B.** Prediction of potential m6A sites of ANLN by SRAMP database. **C.** Putative 4044 m6A site in 3′UTR , with corresponding position and score.


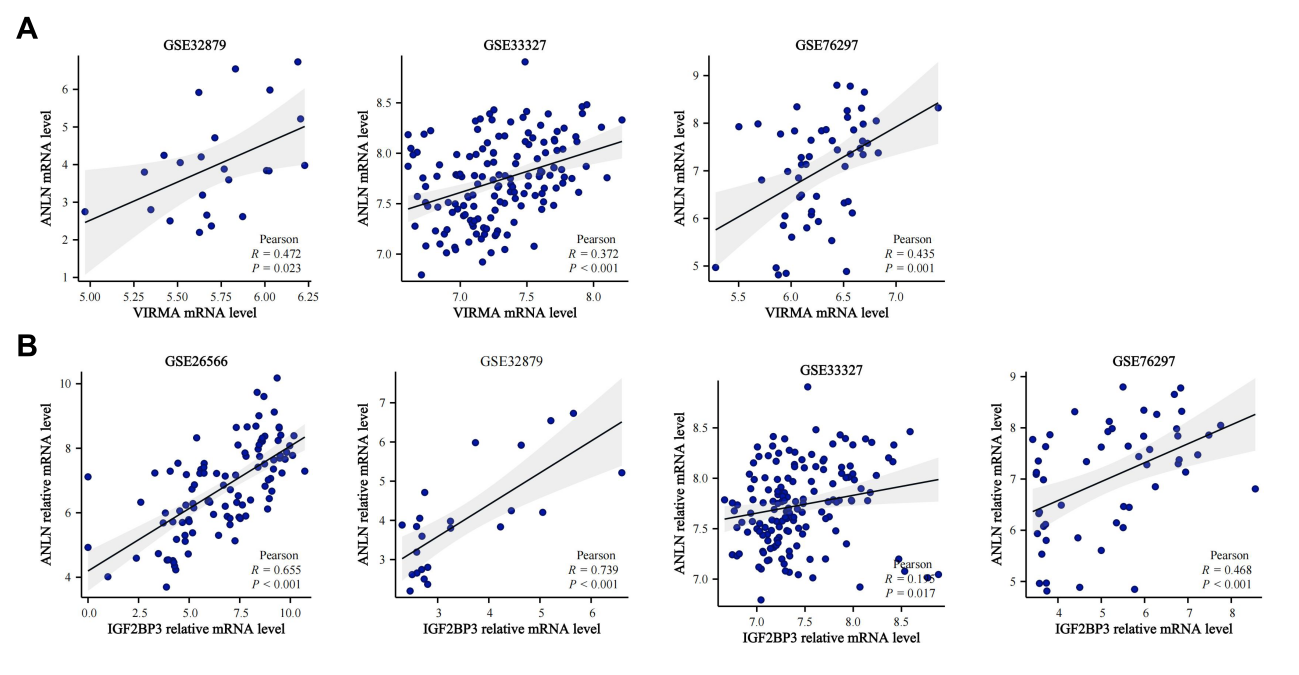


**Figure S4**

**A.** Correlation analysis of VIRMA mRNA level with ANLN mRNA level in GSE32879, GSE33327, GSE76297 data sets. **B.** Correlation analysis of IGF2BP3 mRNA level with ANLN mRNA level in GSE26566, GSE32879, GSE33327, GSE76297 data sets.


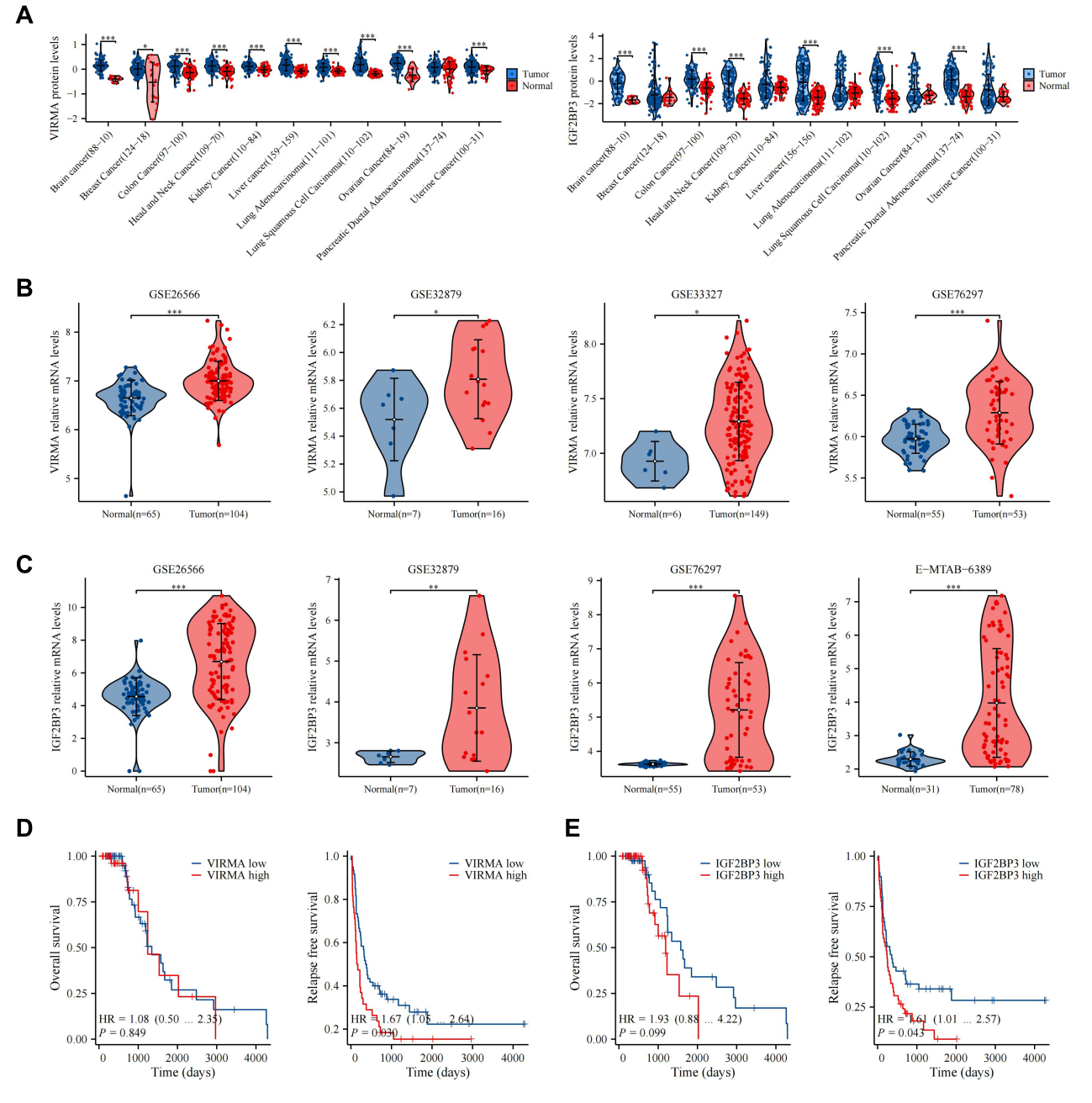


**Figure S5**

1. The protein levels of VIRMA (left) and IGF2BP3 (right) in pan-cancer tissues and adjacent non-tumor tissues from cProSite proteogenomic database. **B.** The mRNA levels of VIRMA in cholangiocarcinoma and non-tumor tissues of four data sets (GSE32879, GSE33327, GSE26566, GSE76297). **C.** The mRNA levels of IGF2BP3 in cholangiocarcinoma and non-tumor tissues of four data sets (GSE32879, GSE26566, GSE76297, E-MTAB-6389). **D.** The overall survival and recurrence free survival of 98 ICC patients were performed according to VIRMA levels. **E.** The overall survival and recurrence free survival of 98 ICC patients were performed according to IGF2BP3 levels. * p<0.05, ** p<0.01, *** p<0.001.


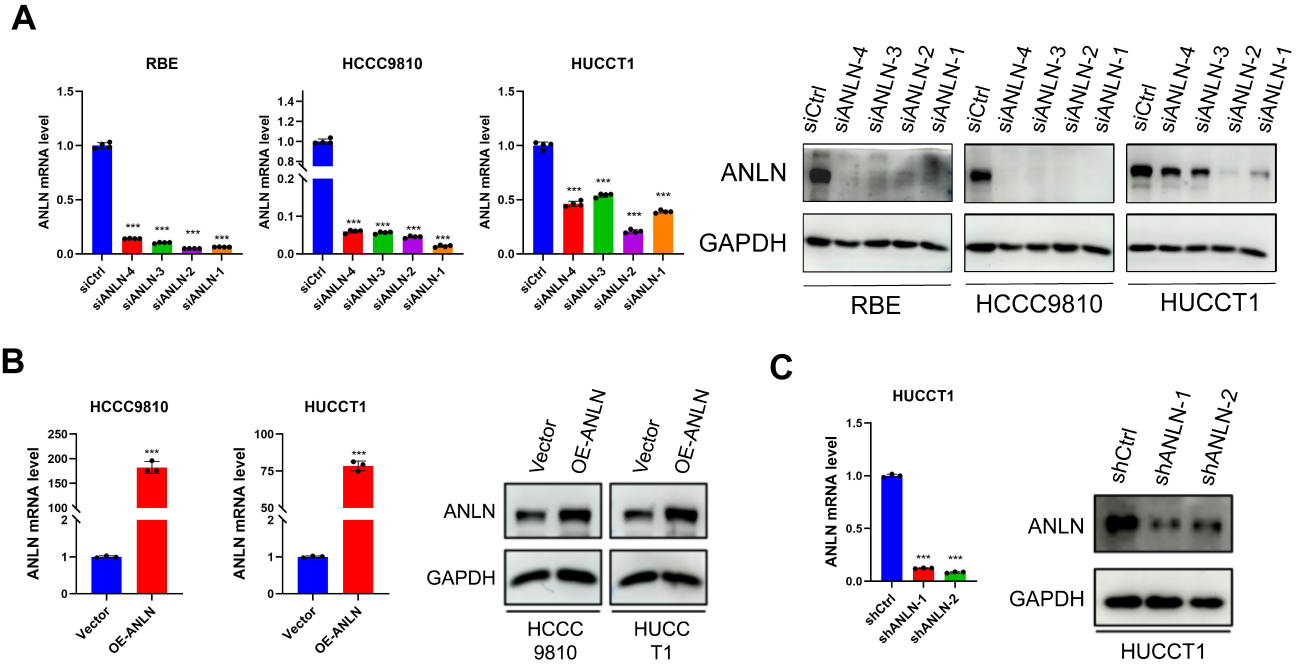


**Figure S6**

**A.** The efficiencies of siANLN was confirmed by qRT‒PCR and western blot. **B.** The efficiencies of overexpression plasmid was confirmed by qRT‒PCR and western blot. **C.** The efficiency of shANLN-constructed HUCCT1 cell line was verified by qRT‒PCR and western blot. n = 3-4. * p<0.05, ** p<0.01, *** p<0.001.


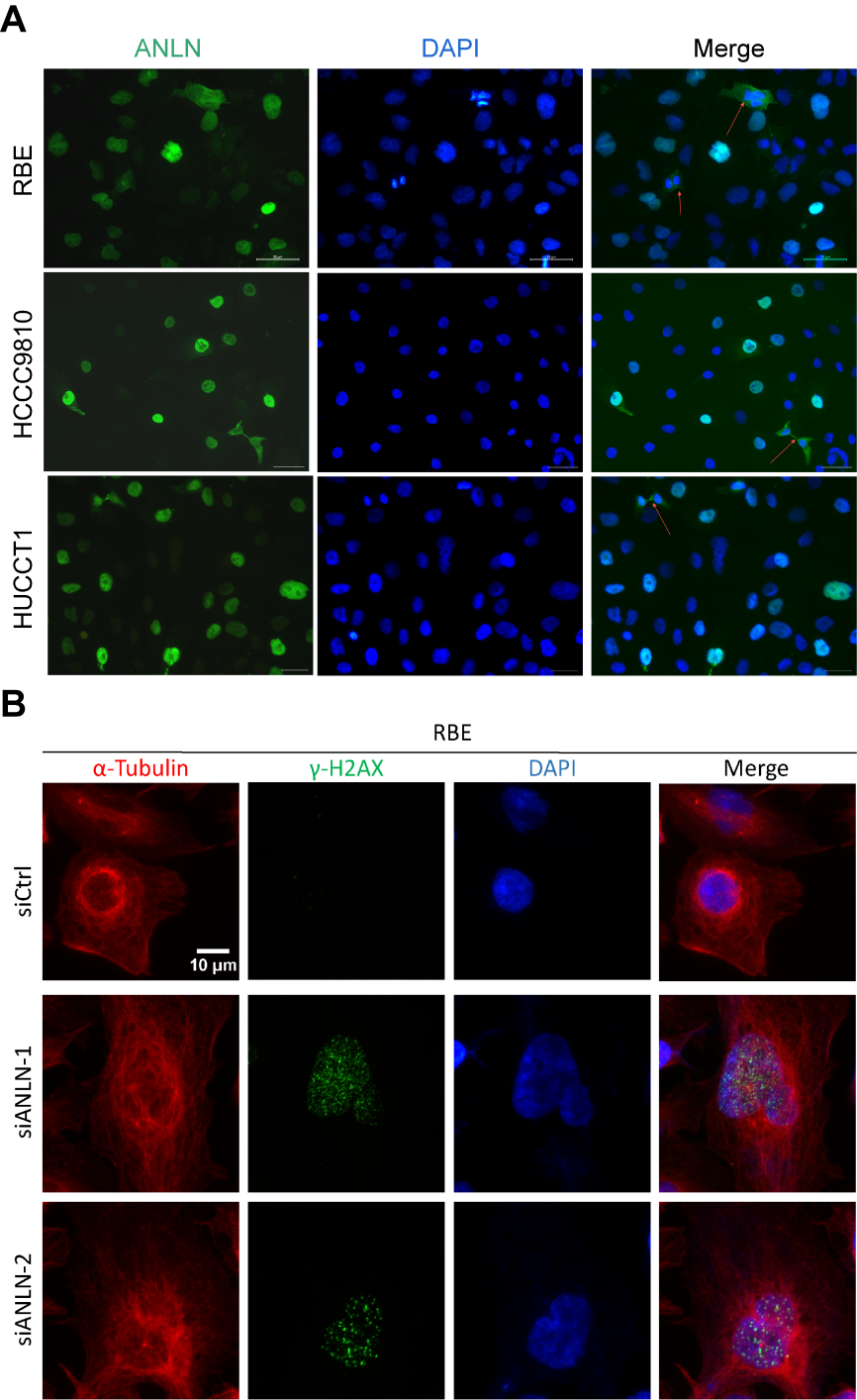


**Figure S7**

1. Immunofluorescence of ANLN (green) and DAPI (blue) in ICC cells. ANLN is localized in the cytoplasm during cytokinesis, with arrow marked. **B.** Representative immunofluorescence images of γH2AX foci (green), α-Tubulin (red) and DAPI (blue) in RBE cells transfected with siANLN and siCtrl, Scale bar = 10μm.

**Figure S8**

1. The mRNA levels of ANLN in HCCC9810 and HUCCT1 cells with the overexpression of vector or YAP5SA. **B.** The protein levels of ANLN in HCCC9810 and HUCCT1 cells with the overexpression of vector or YAP5SA. **C.** Immunofluorescence of YAP1 (red) in siCtrl+vector, siANLN+vector and siANLN+YAP5SA ICC cells, with YAP1 subcellular localization proportion exhibited in the histogram. Scale bar = 10μm. n = 3-4. * p<0.05, ** p<0.01, *** p<0.001.

**
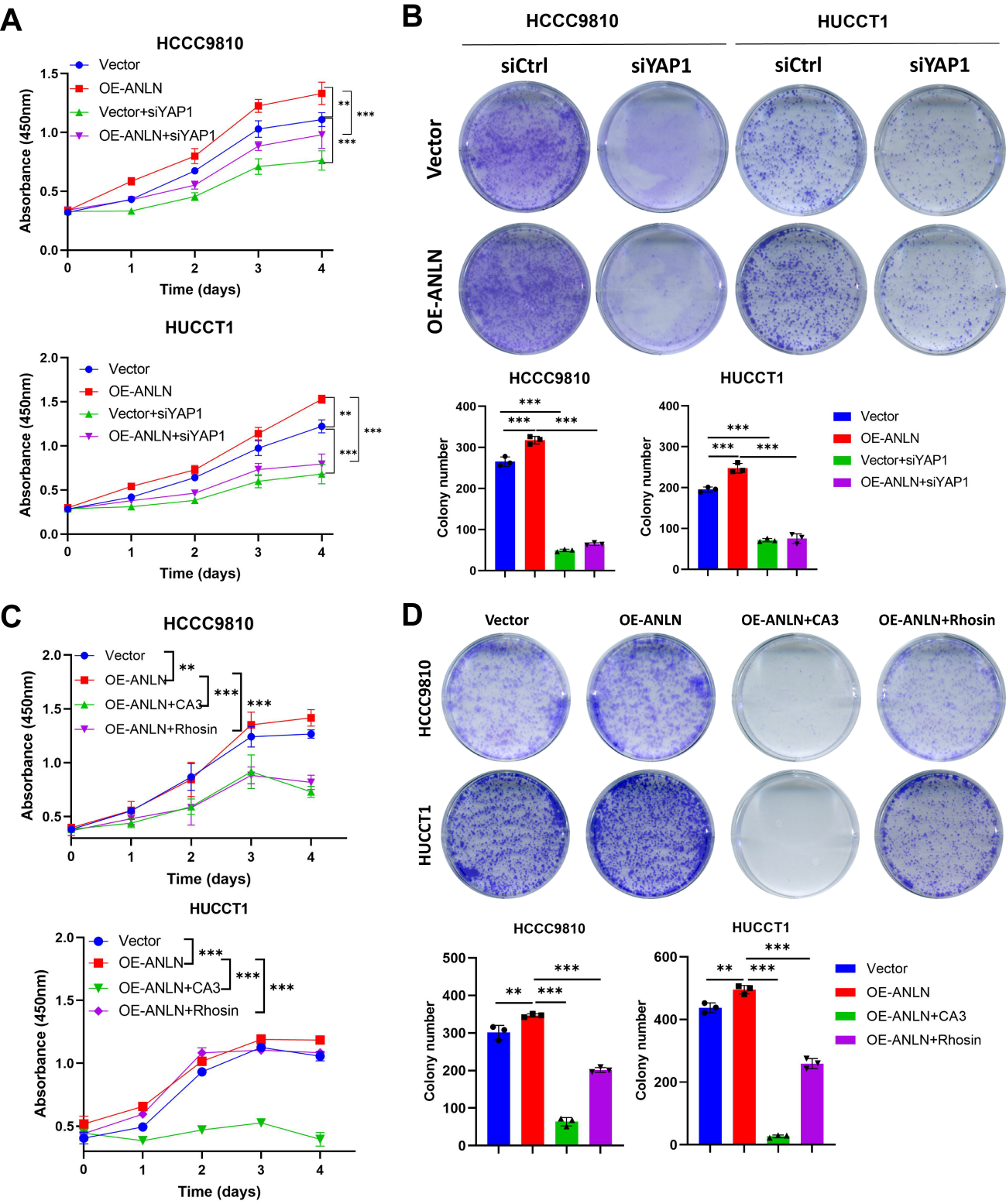
**

**Figure S9**

1. The cck8 growth curves and **B.** colony formation assay in control and ANLN-overexpressing ICC cells with or without YAP1 silencing. **C.** The cck8 growth curves and **D.** Colony formation assay in vector, ANLN-overexpressing ICC cells and ANLN-overexpressing ICC cells treated with CA3 (1 μM) and Rhosin (50 μM). These results are presented as mean values with standard deviation (SD). n=3-6. * p<0.05, ** p<0.01, *** p<0.001.


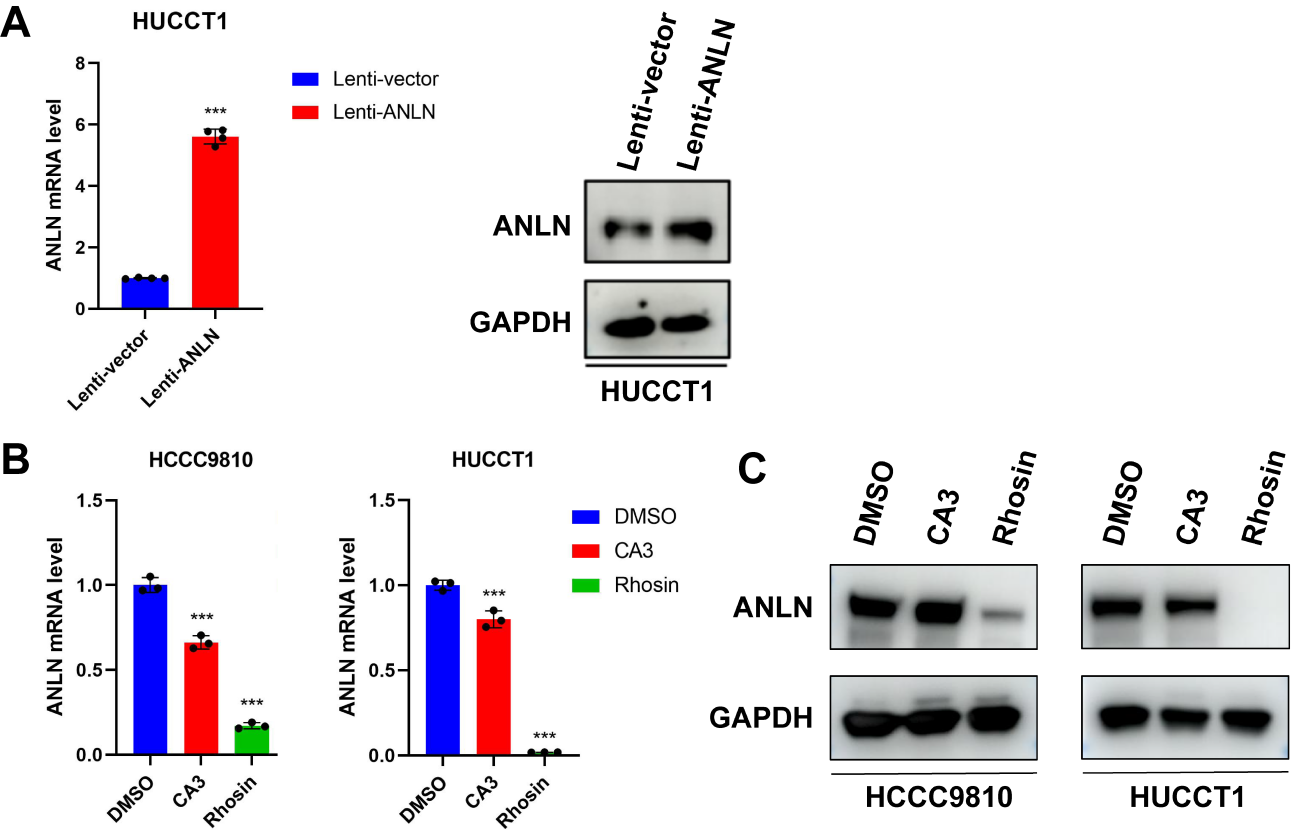


**Figure S10**

**A.** The efficiency of Lenti-ANLN-constructed HUCCT1 cell line was verified by qRT‒PCR and western blot. **B.** The mRNA and **C.** protein level of ANLN in ICC cells exposed to DMSO, CA3 (1 μM) and Rhosin (50 μM). These results are presented as mean values with standard deviation (SD). n = 3-4. * p<0.05, ** p<0.01, *** p<0.001.
