# Supplementary material for: VIRMA/IGF2BP3-mediated ANLN upregulation promotes intrahepatic cholangiocarcinoma growth by forming a positive feedback loop with RhoA/YAP1/TEAD1 signaling pathway
Source: Cell Death Dis. 2026 Jan 9;17(1):20. doi: 10.1038/s41419-025-08197-5 (PMC12789643; doi:10.1038/s41419-025-08197-5)
Supplement: Supplementary file 2 — Supplementary Tables [file 41419_2025_8197_MOESM2_ESM.docx]

**VIRMA/IGF2BP3-mediated ANLN upregulation promotes intrahepatic** **cholangiocarcinoma growth by forming a positive feedback loop with RhoA/YAP1/TEAD1 signaling pathway**

Jiajun Zhang^1^, Ning Huang^1^, Lin-rui Gao^2^, Ai Guo^2^, Hongming Deng^2^, Liming Wang^1,^*****, Mei Liu^2,^*****

^1^Department of Hepatobiliary Surgery, National Cancer Center/National Clinical Research Center for Cancer/Cancer Hospital, Chinese Academy of Medical Sciences and Peking Union Medical College,Beijing, China

^2^Laboratory of Cell and Molecular Biology & State Key Laboratory of Molecular Oncology, National Cancer Center/National Clinical Research Center for Cancer/Cancer Hospital, Chinese Academy of Medical Sciences and Peking Union Medical College, Beijing, China

*** Correspondence should be addressed to:**

Liming Wang

Department of Hepatobiliary Surgery, National Cancer Center/National Clinical Research Center for Cancer/Cancer Hospital, Chinese Academy of Medical Sciences and Peking Union Medical College, 17 Panjiayuan Nanli, Chaoyang District, Beijing 100021, China

Mobile phone: +86-13161361180; Email: [stewen_wang@sina.com](mailto:stewen_wang@sina.com)

Mei Liu, Ph.D

Laboratory of Cell and Molecular Biology & State Key Laboratory of Molecular Oncology, National Cancer Center/National Clinical Research Center for Cancer/Cancer Hospital, Chinese Academy of Medical Sciences and Peking Union Medical College, 17 Panjiayuan Nanli, Chaoyang District, Beijing 100021, China

Tel: +8610 87788487; Fax: +8610 67738220;

E-mail: [liumei@cicams.ac.cn](mailto:liumei@cicams.ac.cn)

**Supplementary Table S1. Clinicopathological characteristics of ICC cohort**

| **characteristics** | **ANLN low** | **ANLN high** | **pvalue** | **statistic** | **method** |
| --- | --- | --- | --- | --- | --- |
| n | 47 | 51 |  |  |  |
| Age, n (%) |  |  | 0.939 | 0.005 | Chisq test |
| ≤60 | 28 (28.6%) | 30 (30.6%) |  |  |  |
| >60 | 19 (19.4%) | 21 (21.4%) |  |  |  |
| Gender, n (%) |  |  | 0.691 | 0.157 | Chisq test |
| male | 23 (23.5%) | 27 (27.6%) |  |  |  |
| female | 24 (24.5%) | 24 (24.5%) |  |  |  |
| Tumor size（cm）, mean ± sd | 5.4 ± 2.3179 | 5.9224 ± 2.7498 | 0.320 | -0.998 | T test |
| Differentiation, n (%) |  |  | 0.572 | 0.319 | Chisq test |
| moderately/well | 22 (22.9%) | 22 (22.9%) |  |  |  |
| poorly | 23 (24%) | 29 (30.2%) |  |  |  |
| Microvascular invasion, n (%) |  |  | 0.440 | 0.595 | Chisq test |
| no | 24 (24.5%) | 30 (30.6%) |  |  |  |
| yes | 23 (23.5%) | 21 (21.4%) |  |  |  |
| Perineural invasion, n (%) |  |  | 0.074 | 3.200 | Chisq test |
| no | 34 (34.7%) | 28 (28.6%) |  |  |  |
| yes | 13 (13.3%) | 23 (23.5%) |  |  |  |
| lymph node metastasis, n (%) |  |  | 0.258 | 1.279 | Chisq test |
| no | 37 (37.8%) | 35 (35.7%) |  |  |  |
| yes | 10 (10.2%) | 16 (16.3%) |  |  |  |

**Supplementary Table S2. The sequences for siRNA**

| **Gene** | **primer** | **sequence** | |
| --- | --- | --- | --- |
| siANLN-1(Human) | Sense (5*′*- 3*′*) | GCUACAUUCUGUUCCCAAATT | |
|  | Anti-sense (5*′*- 3*′*) | UUUGGGAACAGAAUGUAGCTT | |
| siANLN-2(Human) | Sense (5*′*- 3*′*) | GCUACAAACCUAUUGGAAATT | |
|  | Anti-sense (5*′*- 3*′*) | UUUCCAAUAGGUUUGUAGCTT | |
| siVIRMA-1(Human) | Sense (5*′*- 3*′*) | CCAUCAUCUUUAGACCUAATT | |
|  | Anti-sense (5*′*- 3*′*) | UUAGGUCUAAAGAUGAUGGTT | |
| siVIRMA-2(Human) | Sense (5*′*- 3*′*) | GGAGUUGGUUACCUUGCUUTT | |
|  | Anti-sense (5*′*- 3*′*) | AAGCAAGGUAACCAACUCCTT | |
| siIGF2BP1(Human) | Sense (5*′*- 3*′*) | GGCUCAGUAUGGUACAGUATT | |
|  | Anti-sense (5*′*- 3*′*) | UACUGUACCAUACUGAGCCTT | |
| siIGF2BP2(Human) | Sense (5*′*- 3*′*) | | CAUGCCGCAUGAUUCUUGATT |
|  | Anti-sense (5*′*- 3*′*) | | UCAAGAAUCAUGCGGCAUGTT |
| siIGF2BP3(Human) | Sense (5*′*- 3*′*) | | GCUGAGAAGUCGAUUACUATT |
|  | Anti-sense (5*′*- 3*′*) | | UAGUAAUCGACUUCUCAGCTT |
| siYAP1-1(Human) | Sense (5*′*- 3*′*) | | CUGCCACCAAGCUAGAUAATT |
|  | Anti-sense (5*′*- 3*′*) | | UUAUCUAGCUUGGUGGCAGTT |
| siYAP1-2(Human) | Sense (5*′*- 3*′*) | | GAUACCUGAUGAUGUACCUTT |
|  | Anti-sense (5*′*- 3*′*) | | AGGUACAUCAUCAGGUAUCTT |
| siTEAD1(Human) | Sense (5*′*- 3*′*) | | GGCCGAUUUGUAUACCGAATT |
|  | Anti-sense (5*′*- 3*′*) | | UUCGGUAUACAAAUCGGCCTT |
| siTEAD2(Human) | Sense (5*′*- 3*′*) | | CGAAGGAAAUCAAGGGAAATT |
|  | Anti-sense (5*′*- 3*′*) | | UUUCCCUUGAUUUCCUUCGTT |
| siTEAD3(Human) | Sense (5*′*- 3*′*) | | CCUUGCUCUCAAUCUGGAGTT |
|  | Anti-sense (5*′*- 3*′*) | | CUCCAGAUUGAGAGCAAGGUA |
| siTEAD4(Human) | Sense (5*′*- 3*′*) | | UCCUGCACACACGUCUCUUTT |
|  | Anti-sense (5*′*- 3*′*) | | AAGAGACGUGUGUGCAGGAAA |
| siANLN-3(Human) | Sense (5*′*- 3*′*) | | GCAGAUACCAUCAGUGAUUTT |
|  | Anti-sense (5*′*- 3*′*) | | AAUCACUGAUGGUAUCUGCTT |
| siANLN-4(Human) | Sense (5*′*- 3*′*) | | CCAGACCUCUGCUUUCAAATT |
|  | Anti-sense (5*′*- 3*′*) | | UUUGAAAGCAGAGGUCUGGTT |

**Supplementary Table S3. Primer List.**

| **Gene** | **Forward primer (5’-3’)** | **Reverse primer (5’-3’)** | |
| --- | --- | --- | --- |
| GAPDH(Human) | Forward (5*′*- 3*′*) | GAGCCAAAAGGGTCATCATCT | |
|  | Reverse (5*′*- 3*′*) | TTCCACGATACCAAAGTTGTCA | |
| ANLN(Human) | Forward (5*′*- 3*′*) | CTGATGATGCGTCTTTGGTT | |
|  | Reverse (5*′*- 3*′*) | GGATGGAACTGTCTGGGATAA | |
| CYR61(Human) | Forward (5*′*- 3*′*) | AGCCTCGCATCCTATACAACC | |
|  | Reverse (5*′*- 3*′*) | TTCTTTCACAAGGCGGCACTC | |
| CTGF(Human) | Forward (5*′*- 3*′*) | AAAAGTGCATCCGTACTCCCA | |
|  | Reverse (5*′*- 3*′*) | CCGTCGGTACATACTCCACAG | |
| ANKRD1(Human) | Forward (5*′*- 3*′*) | CACTTCTAGCCCACCCTGTGA | |
|  | Reverse (5*′*- 3*′*) | CCACAGGTTCCGTAATGATTT | |
| ANLN(Human)  MeRIP-primer | Forward (5*′*- 3*′*) | | TGTCTAAGGCCTTTACAGAAACAT |
|  | Reverse (5*′*- 3*′*) | | TGAACCCCCAAGTCTCACAC |
| ANLN(Human)  Chip-primer 1 | Forward (5*′*- 3*′*) | | TCCTCCCGCTCTAACACTGTAC |
|  | Reverse (5*′*- 3*′*) | | TCCCCGACACTCTAAGCCAATG |
| ANLN(Human)  Chip-primer 2 | Forward (5*′*- 3*′*) | | AAGGGCTCTTTGTCTGGCTCTG |
|  | Reverse (5*′*- 3*′*) | | GGAGGCTGAGGCAGGAGAATTG |
| ANLN(Human)  Chip-primer 3 | Forward (5*′*- 3*′*) | | AAGGCGGGAACACCCAAACG |
|  | Reverse (5*′*- 3*′*) | | CGCTTCCTGCTCTCCAACGG |

**Supplementary Table S4. Antibody List.**

| **Antigens** | **Manufacturer** | **Application** |
| --- | --- | --- |
| ANLN | Abcam (ab211872), USA | 1:1000 for WB or 1:50 for IHC or 1:1000 for IF |
| VIRMA | Proteintech Group ( 25712-1-AP), China | 1:1000 for WB or 1:200 for IHC or 1:1000 for IF |
| IGF2BP3 | Proteintech Group (14642-1-AP), China | 1:1000 for WB or 1:100 for IHC or 1:1000 for IF |
| YAP1 | Cell Signaling Technology (#14074), Beverly, MA | 1:1000 for WB or  1:1000 for IF or  Chip for 1:100 |
| TEAD1 | Cell Signaling Technology (#12292), Beverly, MA | 1:1000 for WB or  Chip for 1:100 |
| LATS1 | Proteintech Group (17049-1-AP), China | 1:1000 for WB |
| Phospho-YAP1 | Cell Signaling Technology (#13008), Beverly, MA | 1:1000 for WB |
| Phospho-LATS1 | Cell Signaling Technology ( #8654), Beverly, MA | 1:1000 for WB |
| α-Tubulin | Abcam (ab4074), USA | 1:1000 for IF |
| γ-H2AX | Merck (JBW301), USA | 1:1000 for WB or 1:1000 for IF |
| Caspase-3 | Cell Signaling Technology (#9665), Beverly, MA | 1:1000 for WB |
| GAPDH | Abmart (M20006) | 1:5000 for WB |
