# Supplementary material for: VIRMA/IGF2BP3-mediated ANLN upregulation promotes intrahepatic cholangiocarcinoma growth by forming a positive feedback loop with RhoA/YAP1/TEAD1 signaling pathway
Source: Cell Death Dis. 2026 Jan 9;17(1):20. doi: 10.1038/s41419-025-08197-5 (PMC12789643; doi:10.1038/s41419-025-08197-5)
Supplement: Supplementary file 3 — Original western blot images [file 41419_2025_8197_MOESM3_ESM.pdf]

Fig1

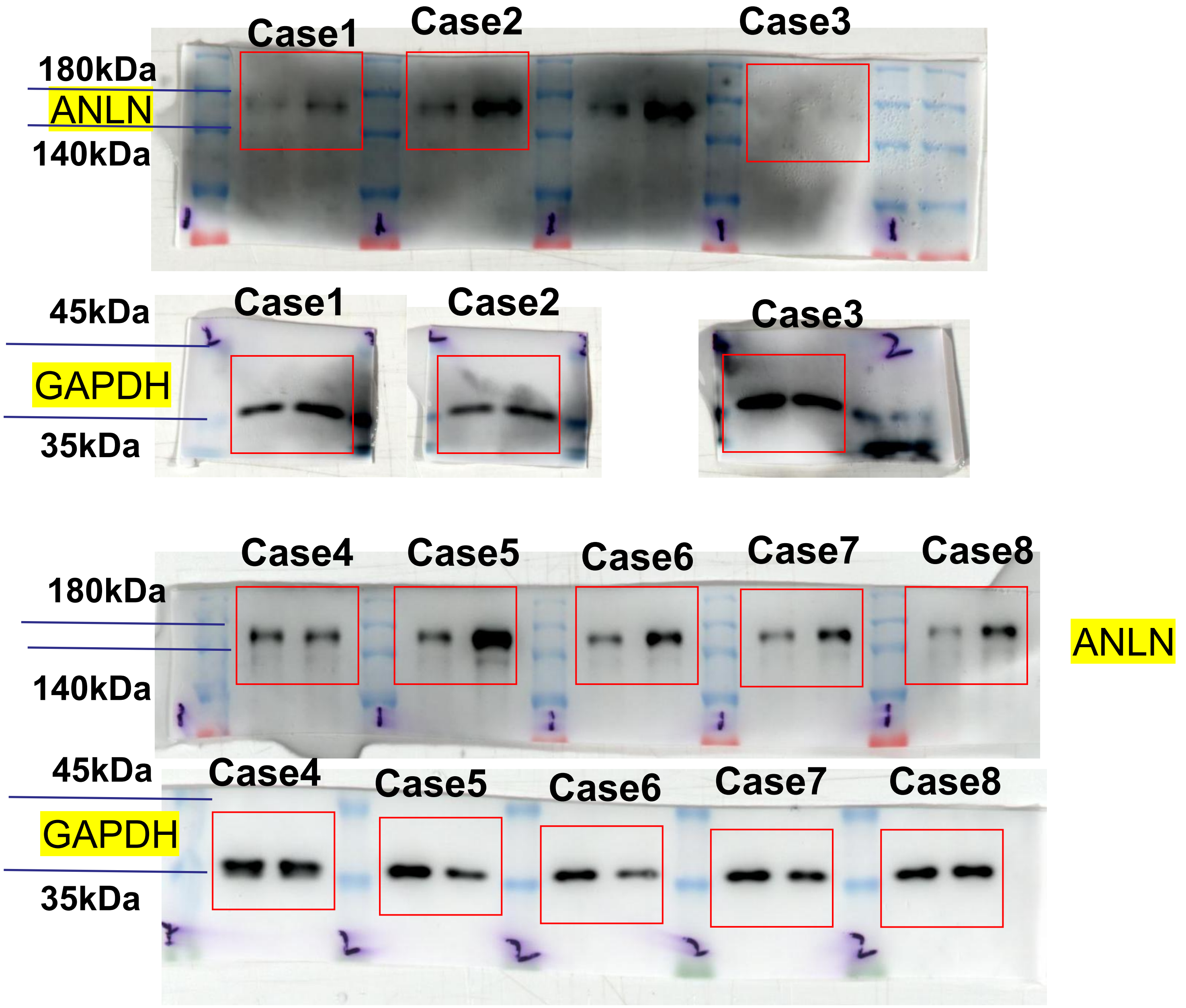
Case1
Case2
Case3
180kDa
ANLN
140kDa
45kDa
GAPDH
35kDa
Case4
Case5
Case6
Case7
Case8
180kDa
140kDa
ANLN
45kDa
GAPDH
35kDa

Fig2

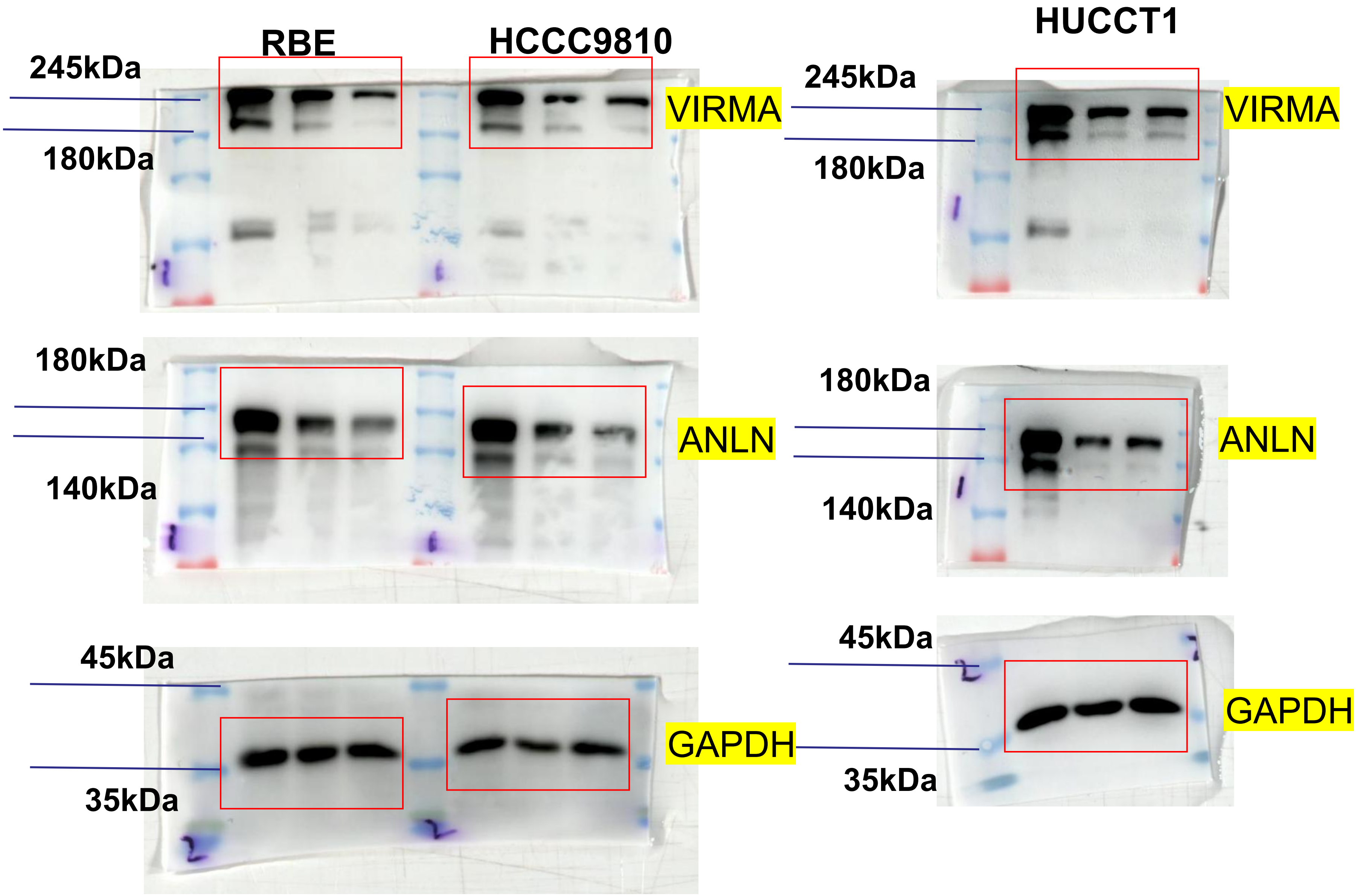
RBE
HCCC9810
HUCCT1
245kDa
180kDa
VIRMA
245kDa
180kDa
VIRMA
180kDa
140kDa
ANLN
180kDa
140kDa
ANLN
45kDa
35kDa
GAPDH
45kDa
35kDa
GAPDH

Fig3

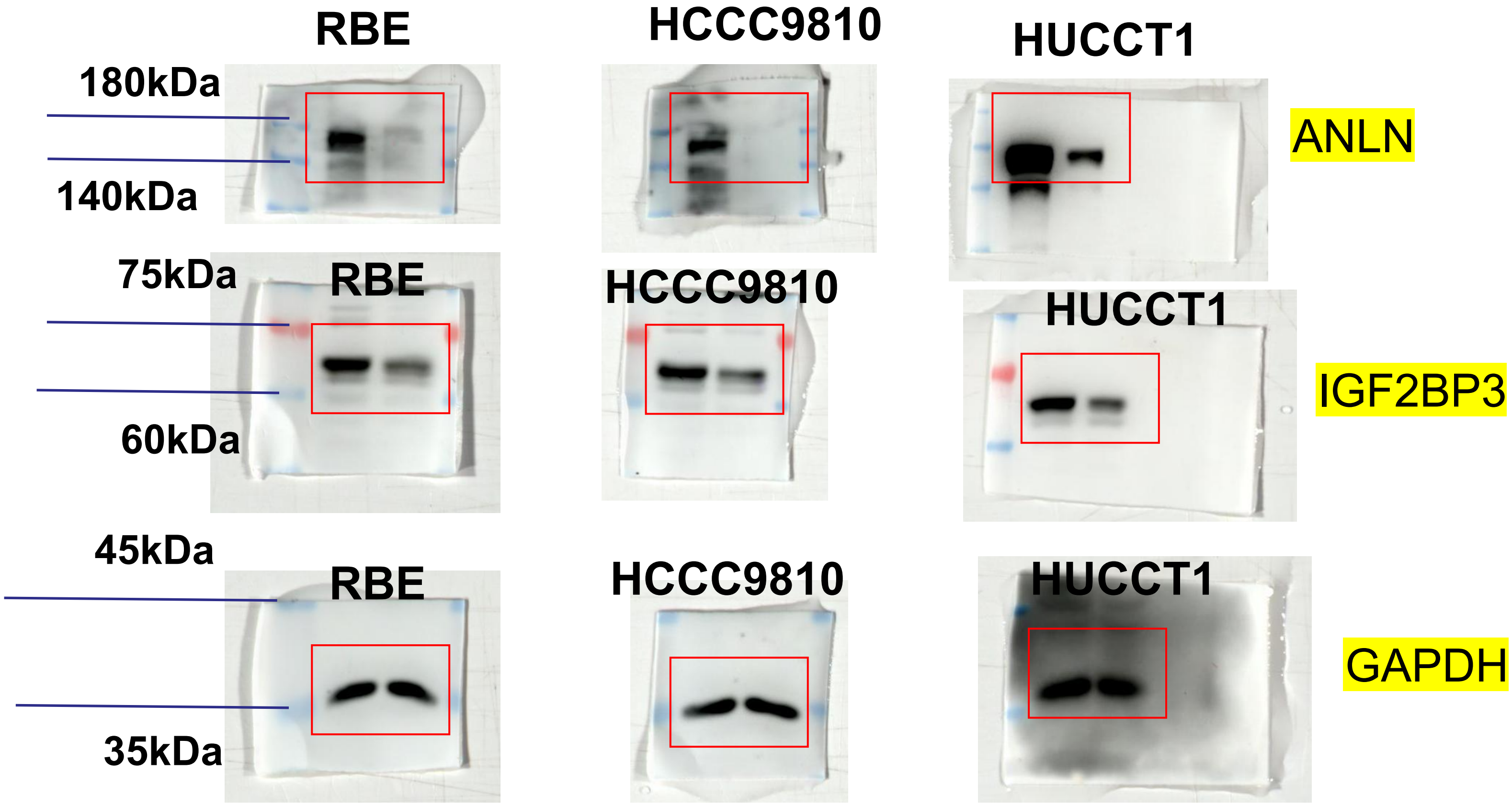

RBE
HCCC9810
HUCCT1
180kDa
140kDa
ANLN
75kDa
60kDa
IGF2BP3
45kDa
35kDa
GAPDH

Fig4

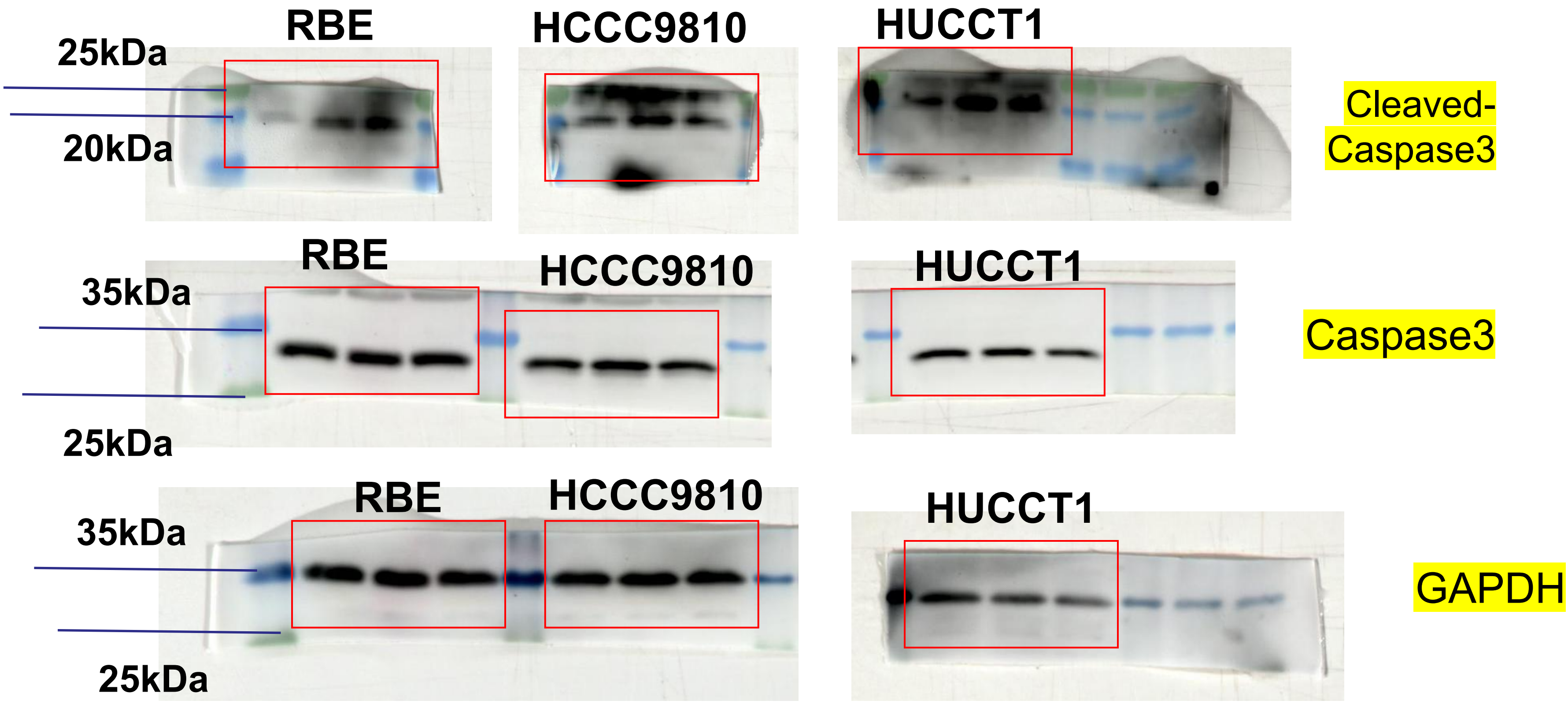
RBE
HCCC9810
HUCCT1
25kDa
20kDa
Cleaved-Caspase3
RBE
HCCC9810
HUCCT1
35kDa
25kDa
Caspase3
RBE
HCCC9810
HUCCT1
35kDa
25kDa
GAPDH

Fig5

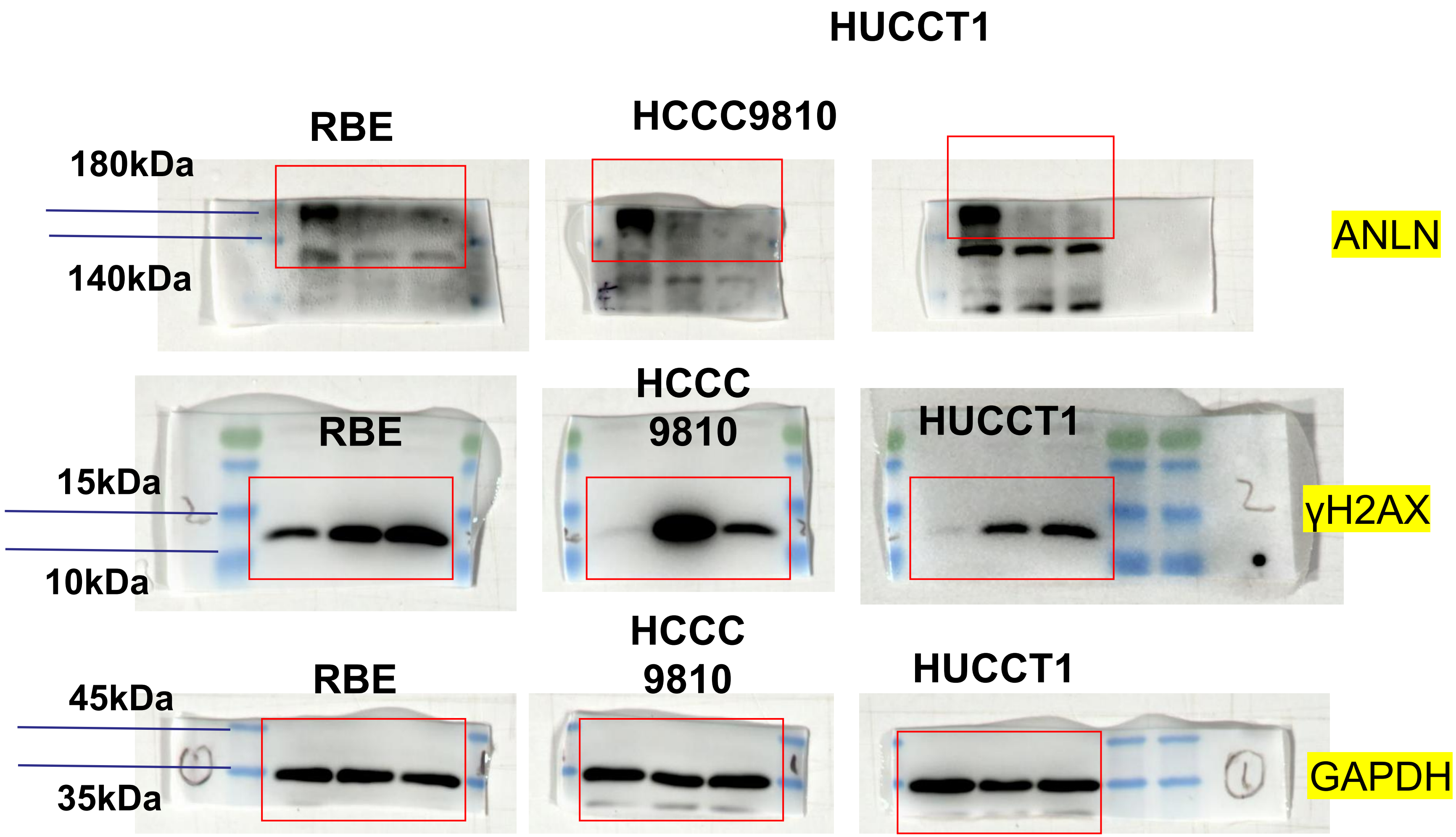
HUCCT1
RBE
HCCC9810
180kDa
140kDa
ANLN
RBE
HCCC
9810
HUCCT1
15kDa
10kDa
γH2AX
RBE
HCCC
9810
HUCCT1
45kDa
35kDa
GAPDH

Fig6B

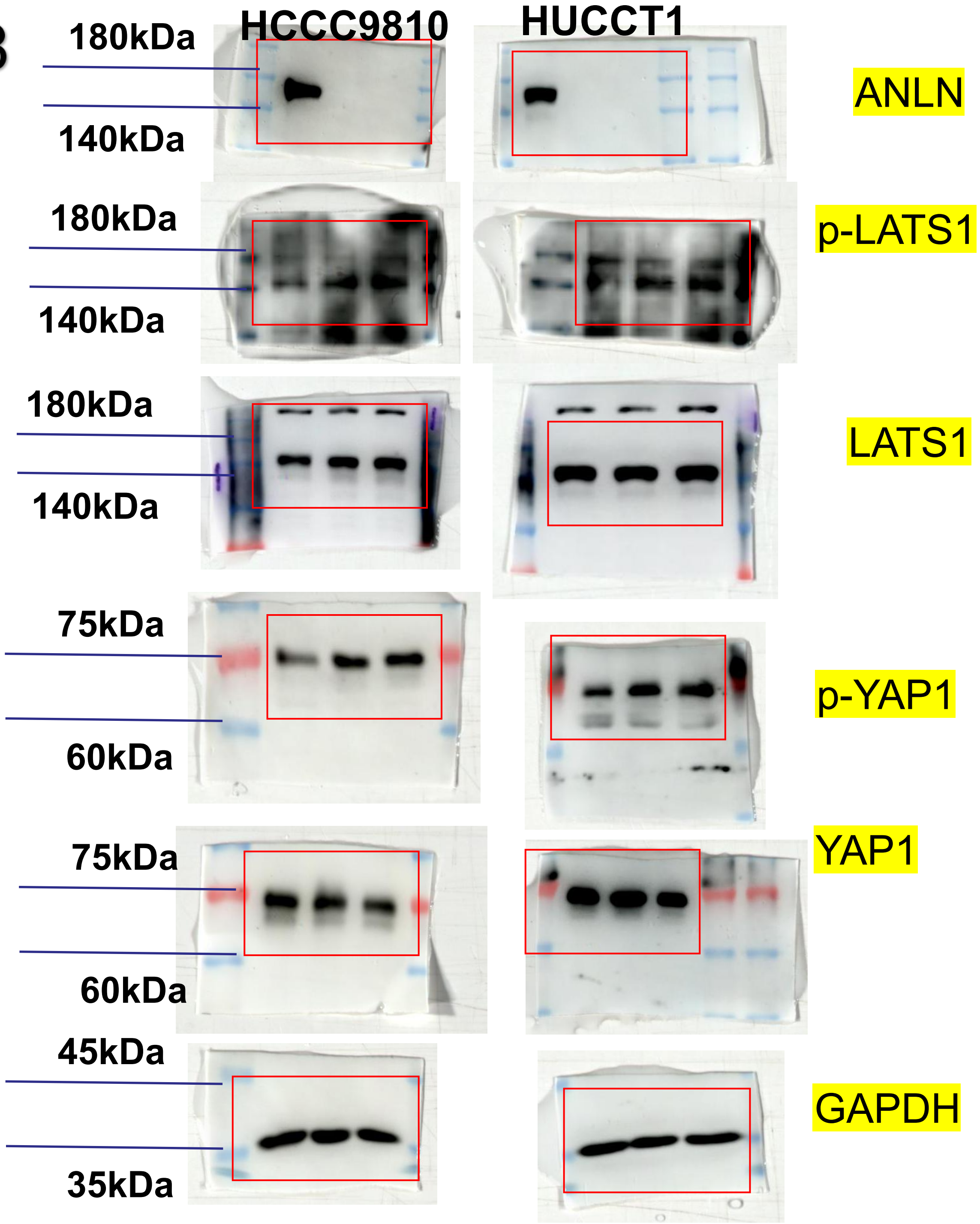
HCCC9810
HUCCT1
180kDa
140kDa
ANLN
180kDa
140kDa
p-LATS1
180kDa
140kDa
LATS1
75kDa
60kDa
p-YAP1
75kDa
60kDa
YAP1
45kDa
35kDa
GAPDH

Fig6C

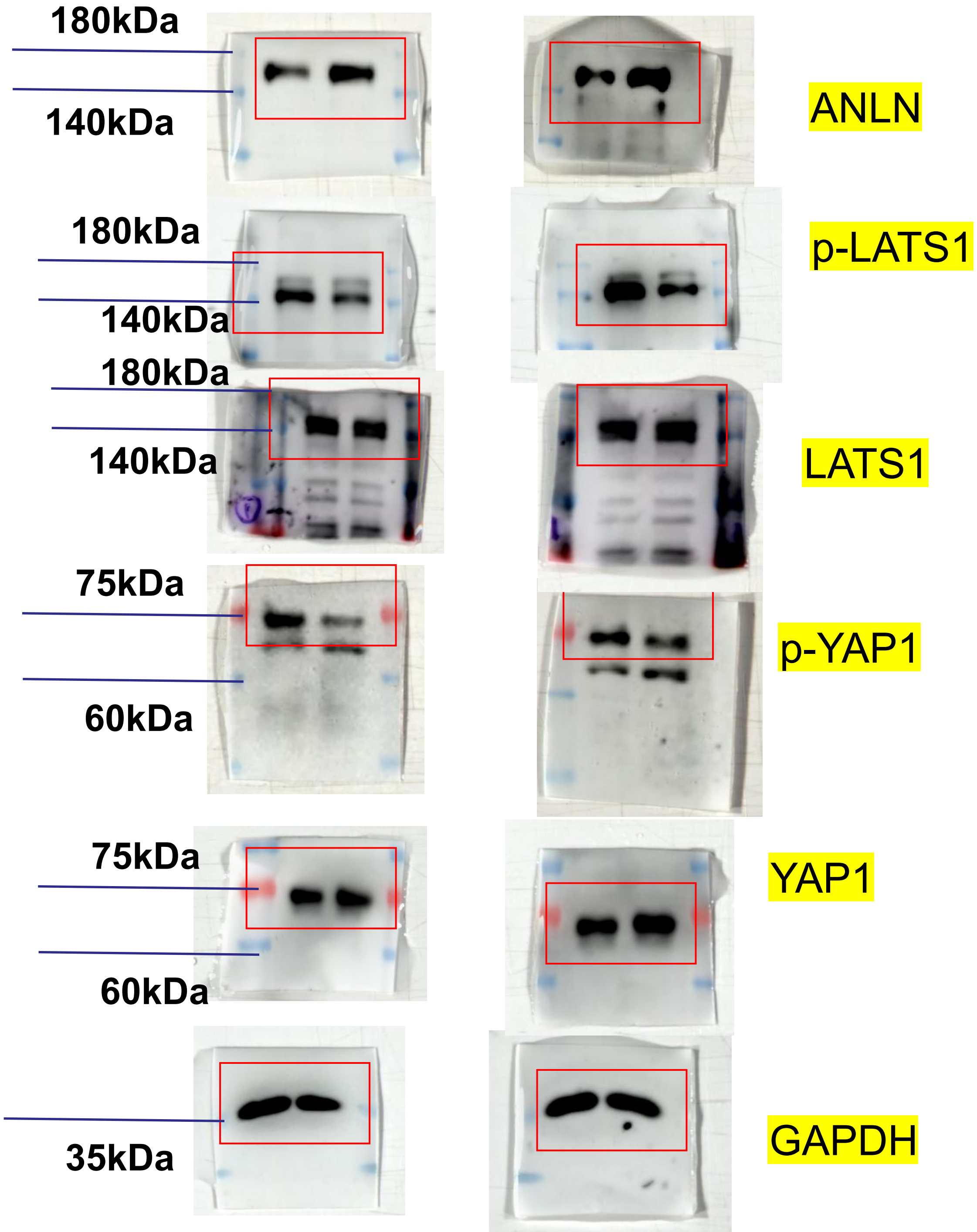
180kDa
140kDa
ANLN
180kDa
140kDa
p-LATS1
180kDa
140kDa
LATS1
75kDa
60kDa
p-YAP1
75kDa
60kDa
YAP1
35kDa
GAPDH

Fig6I

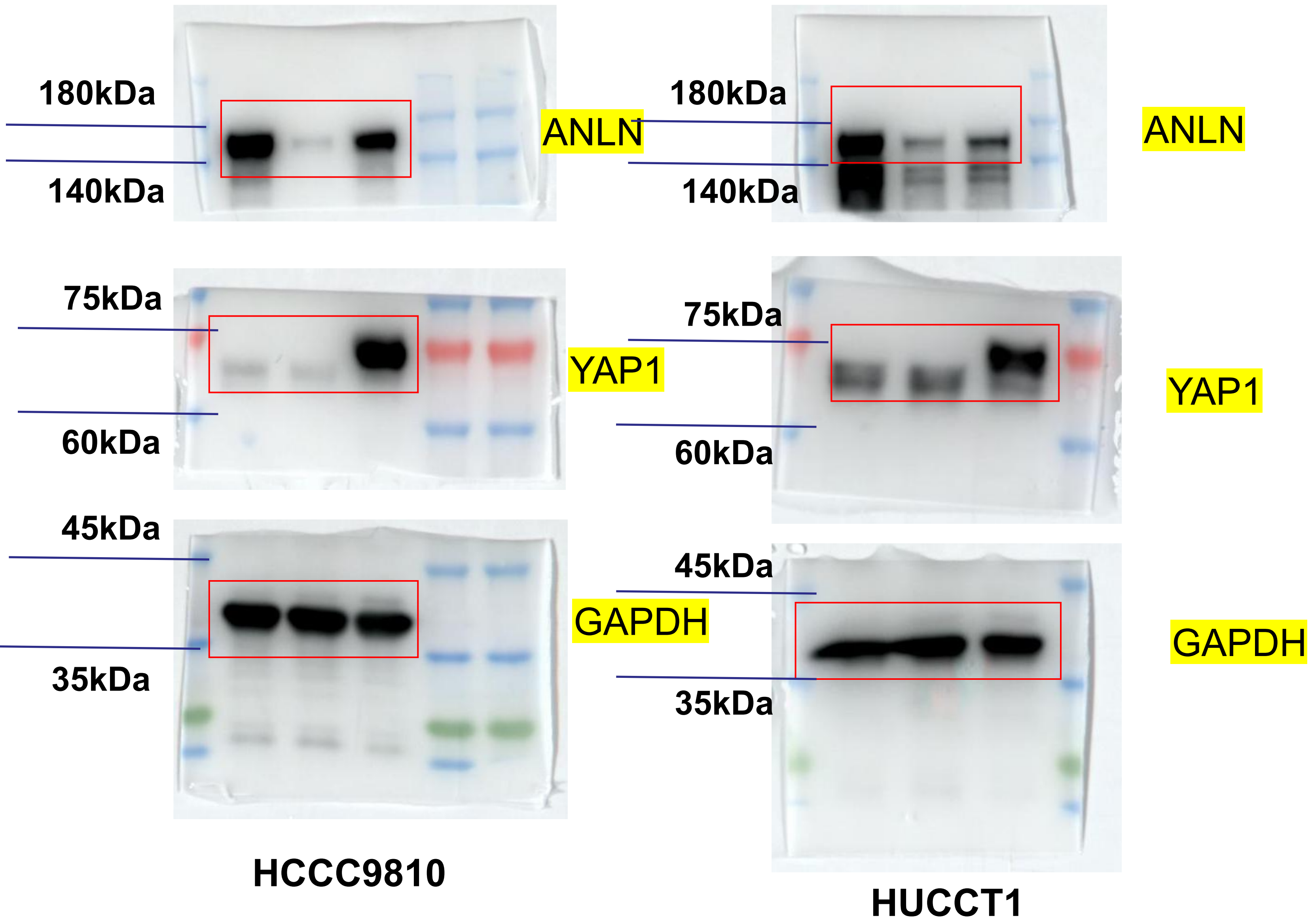
180kDa
140kDa
ANLN
75kDa
60kDa
YAP1
45kDa
35kDa
GAPDH
HCCC9810
180kDa
140kDa
ANLN
75kDa
60kDa
YAP1
45kDa
35kDa
GAPDH
HUCCT1

Fig7C

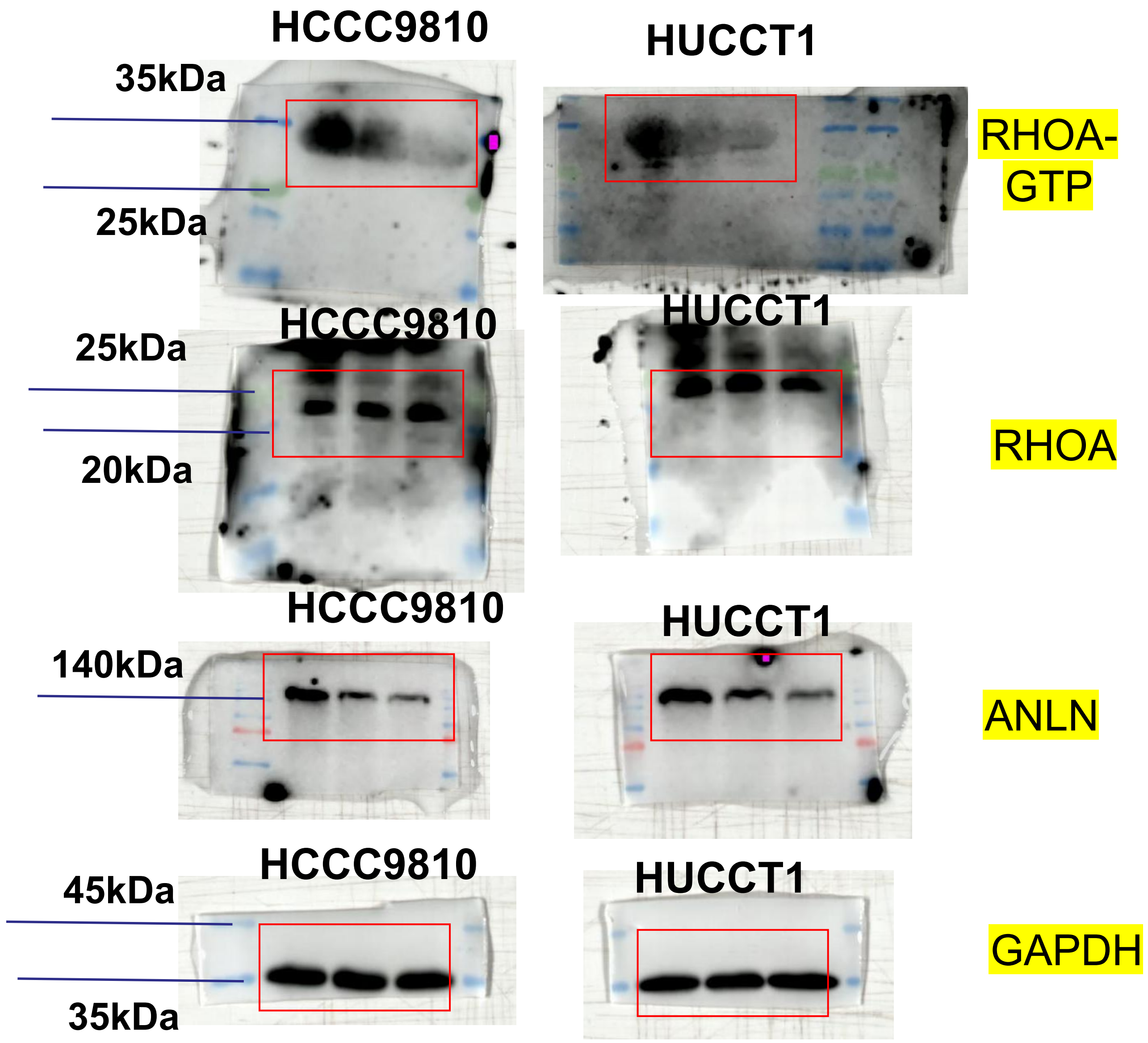

HCCC9810
HUCCT1
35kDa
25kDa
RHOA-GTP
HCCC9810
HUCCT1
25kDa
20kDa
RHOA
HCCC9810
HUCCT1
140kDa
ANLN
HCCC9810
HUCCT1
45kDa
35kDa
GAPDH

Fig7D

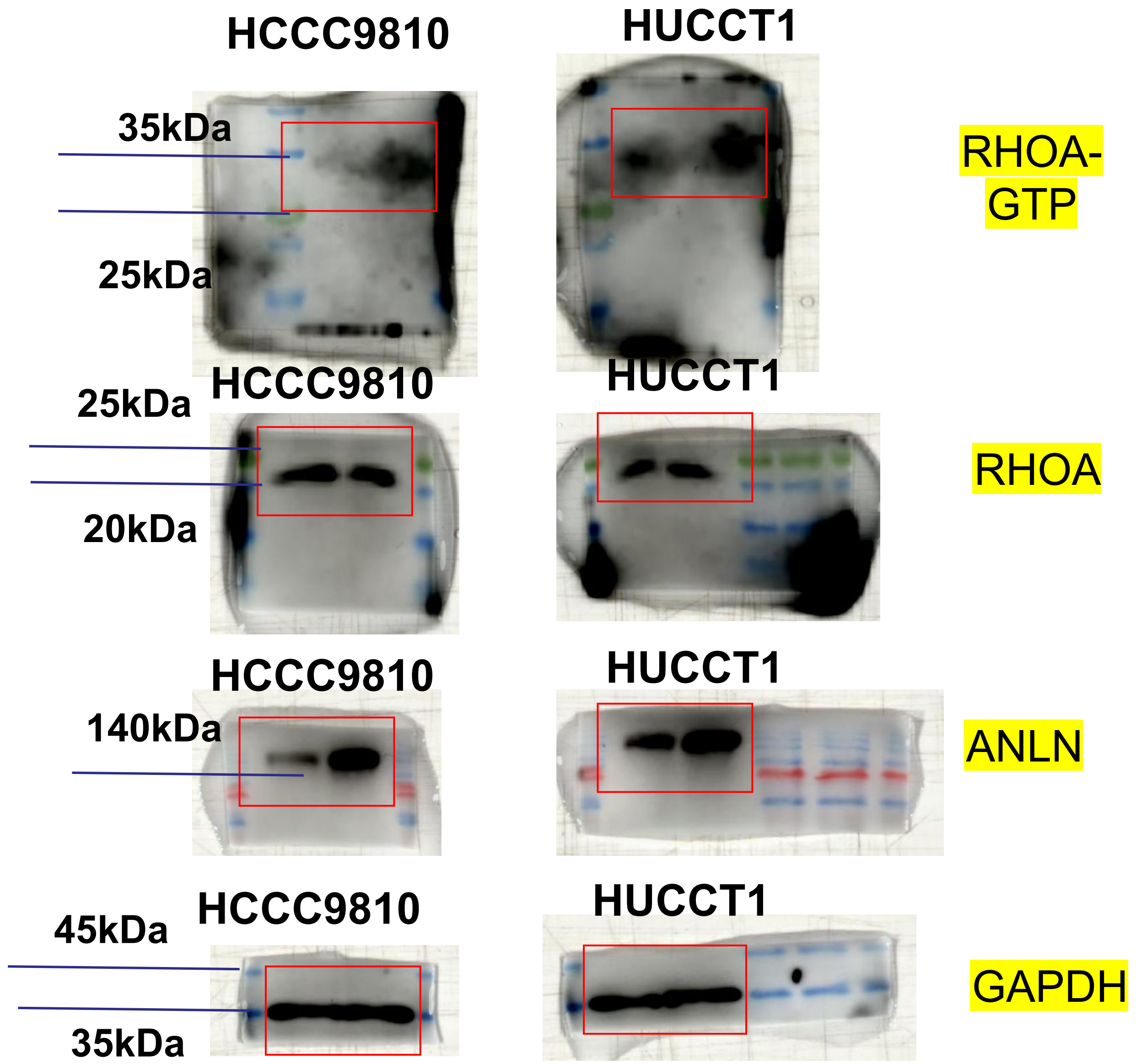
HCCC9810
HUCCT1
35kDa
25kDa
RHOA-GTP
HCCC9810
HUCCT1
25kDa
20kDa
RHOA
HCCC9810
HUCCT1
140kDa
ANLN
45kDa
HCCC9810
HUCCT1
35kDa
GAPDH

Fig7E

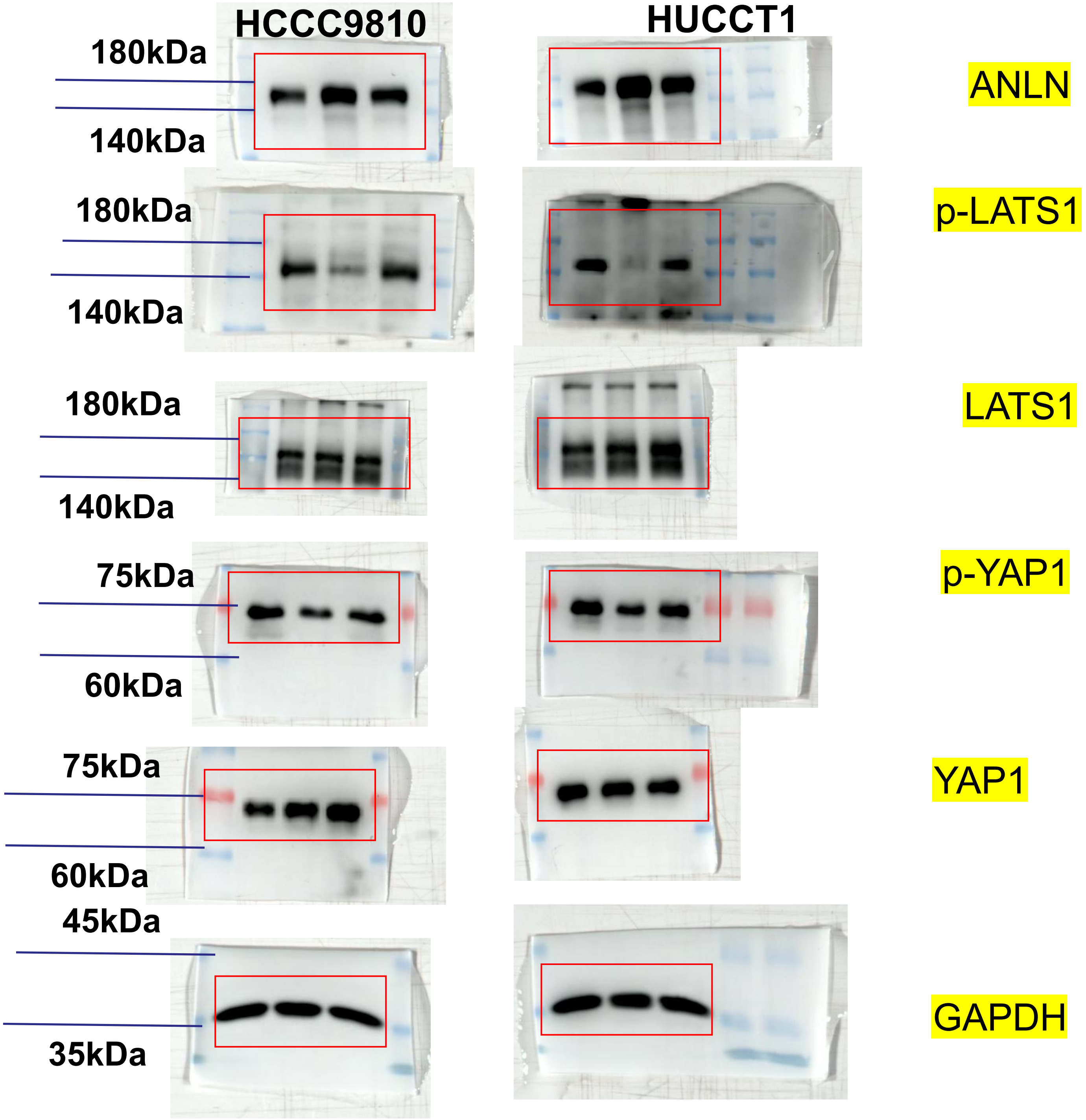
HCCC9810
HUCCT1
180kDa
140kDa
180kDa
140kDa
180kDa
140kDa
75kDa
60kDa
75kDa
60kDa
45kDa
35kDa
ANLN
p-LATS1
LATS1
p-YAP1
YAP1
GAPDH

Fig8C

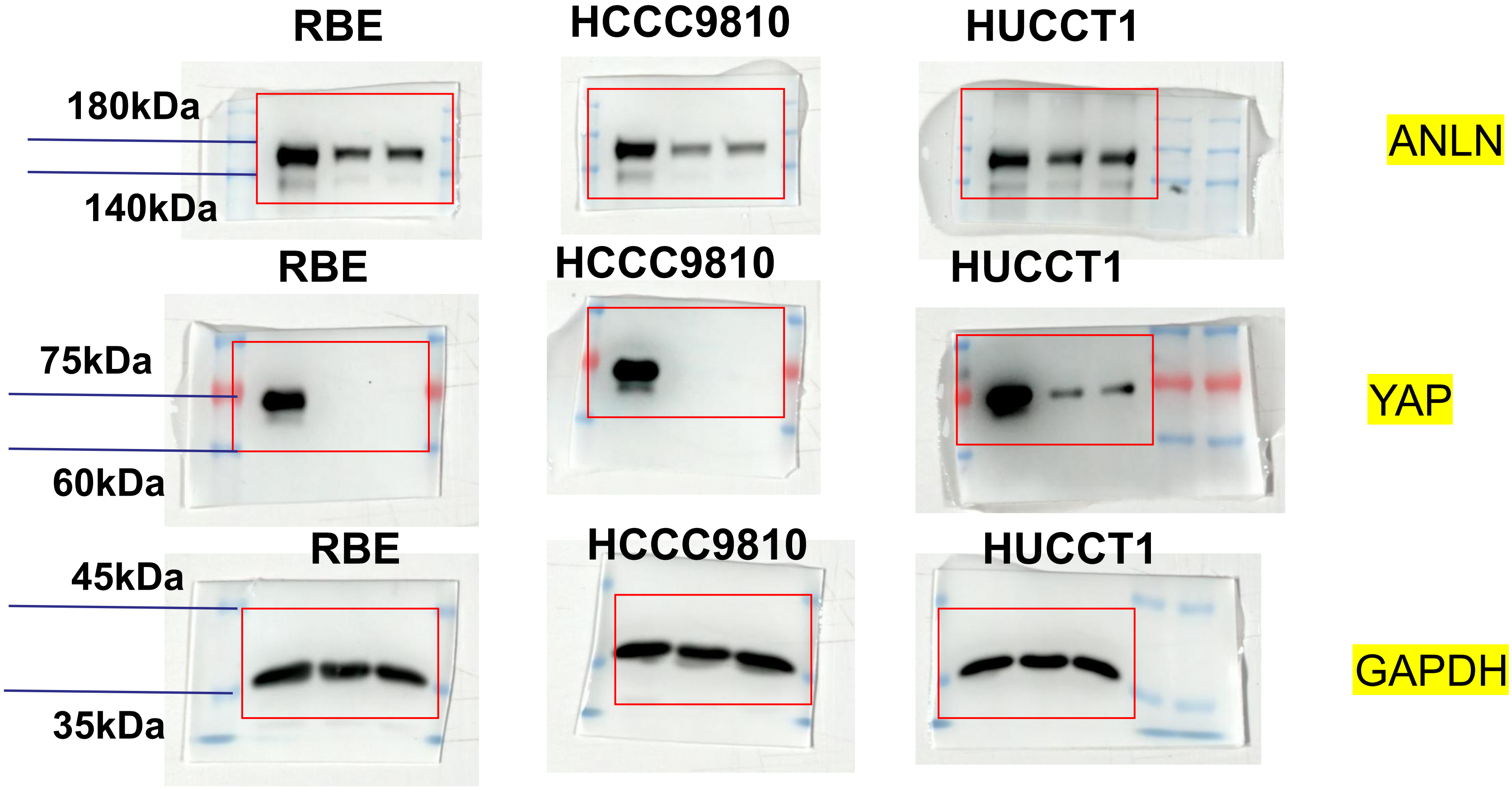
RBE
HCCC9810
HUCCT1
180kDa
140kDa
ANLN
RBE
HCCC9810
HUCCT1
75kDa
60kDa
YAP
RBE
HCCC9810
HUCCT1
45kDa
35kDa
GAPDH

Fig8E

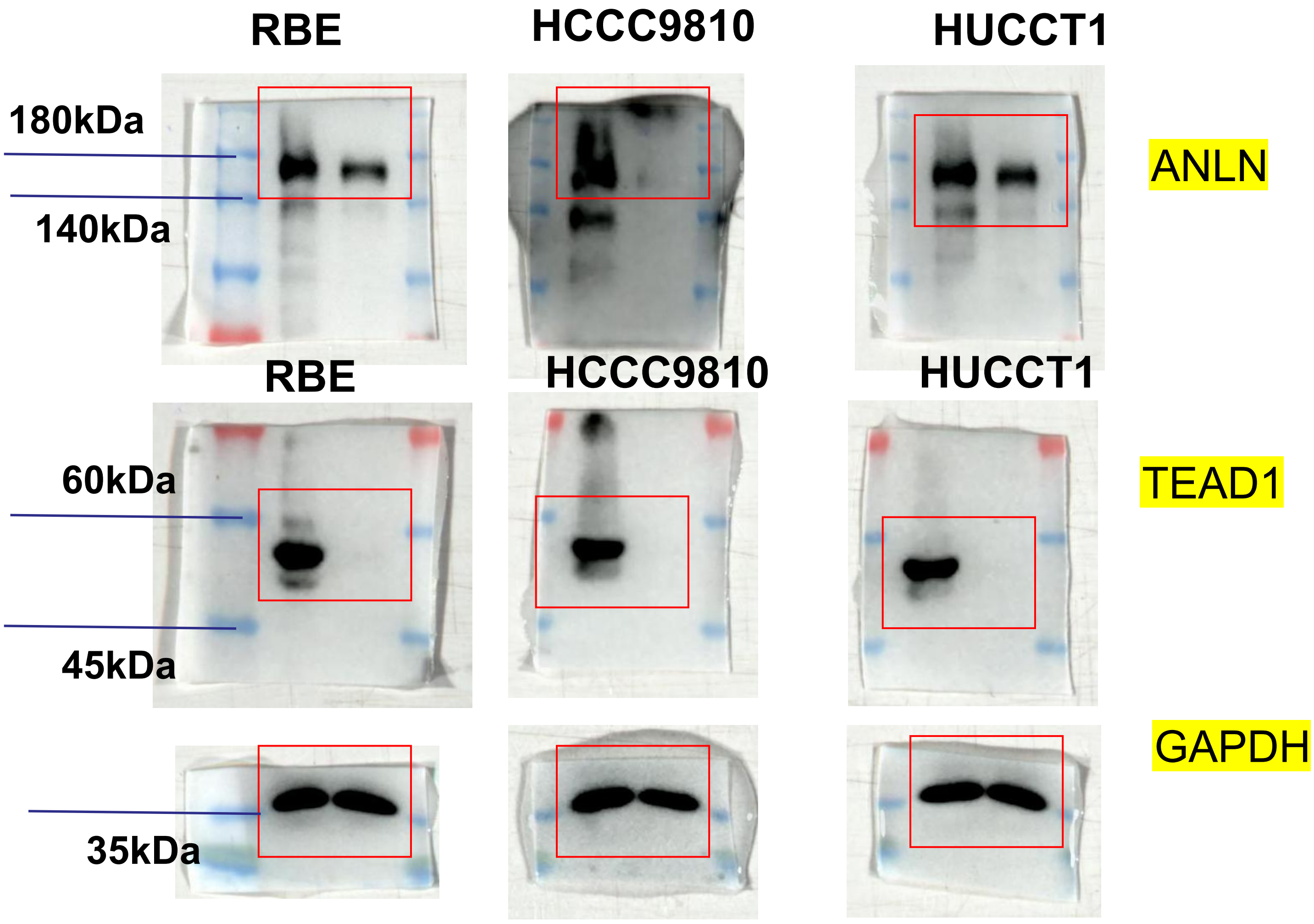

RBE
HCCC9810
HUCCT1
180kDa
140kDa
ANLN
RBE
HCCC9810
HUCCT1
60kDa
45kDa
TEAD1
35kDa
GAPDH

Fig8L

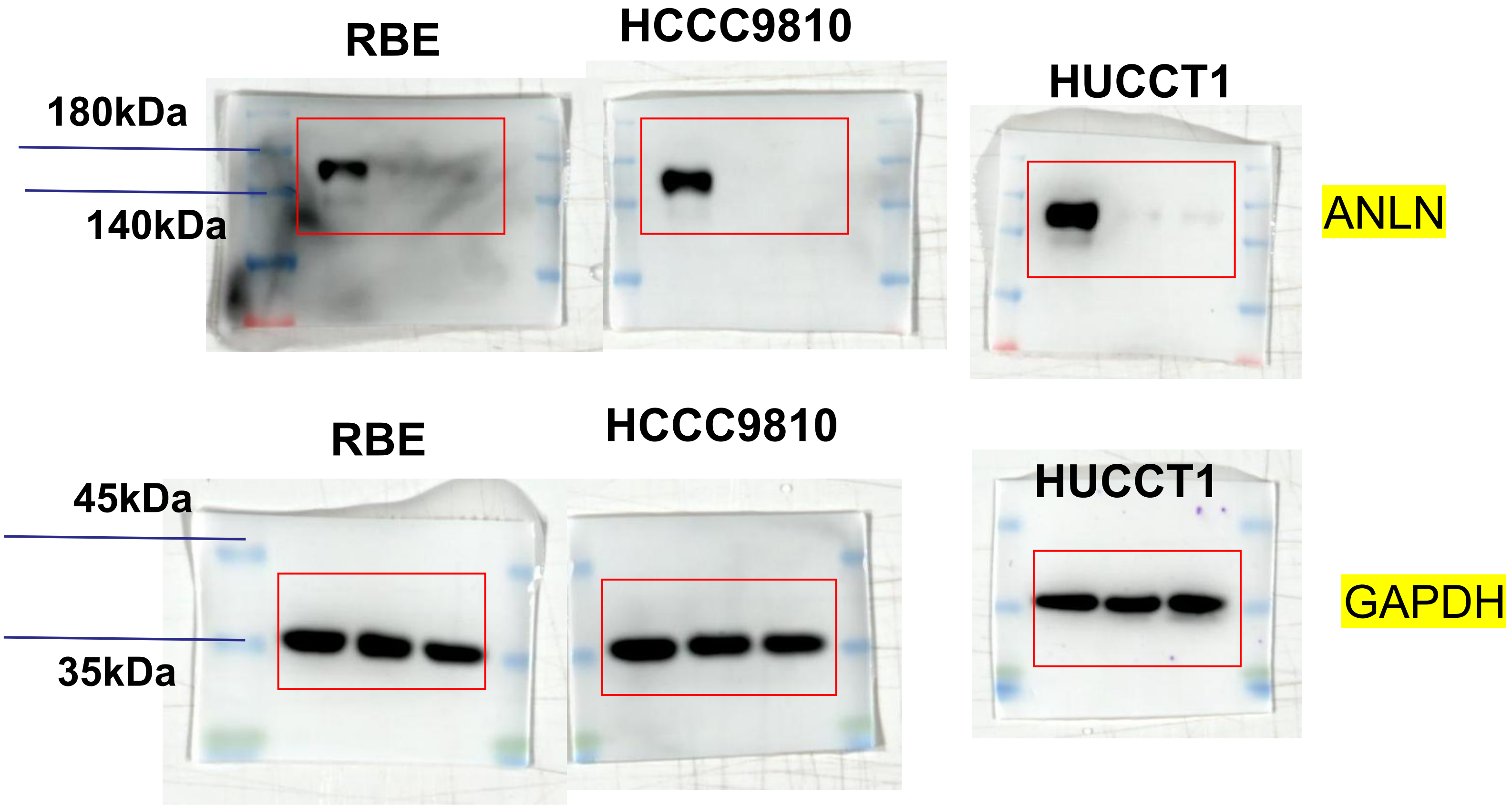

RBE
HCCC9810
HUCCT1
180kDa
140kDa
ANLN
RBE
HCCC9810
HUCCT1
45kDa
35kDa
GAPDH

FigS6A

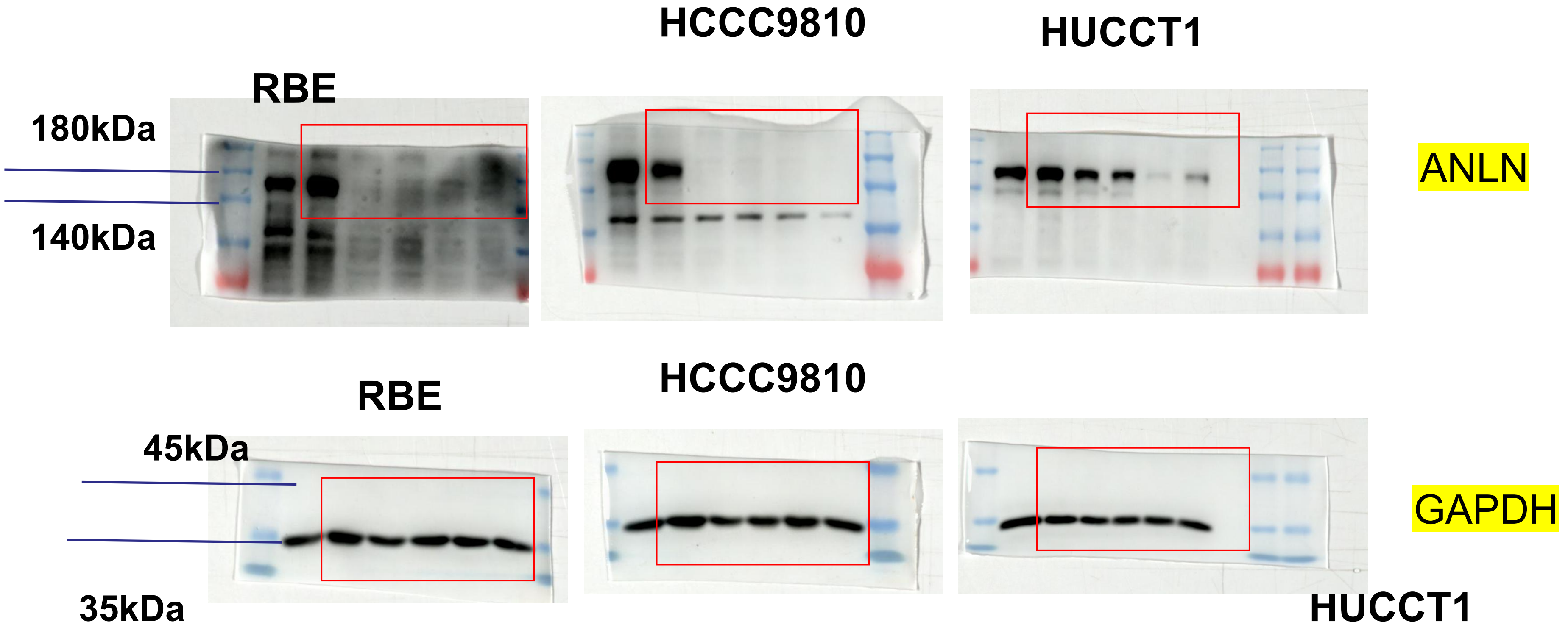
HCCC9810
HUCCT1
RBE
180kDa
140kDa
ANLN
RBE
HCCC9810
45kDa
GAPDH
35kDa
HUCCT1

FigS6B

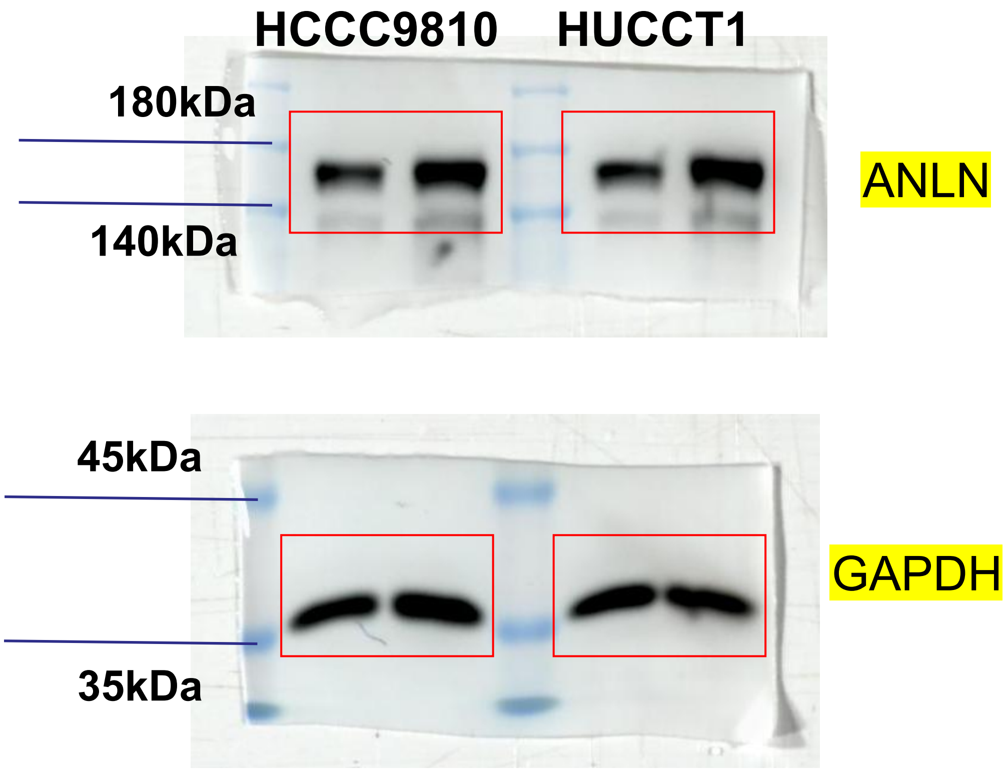

FigS6C

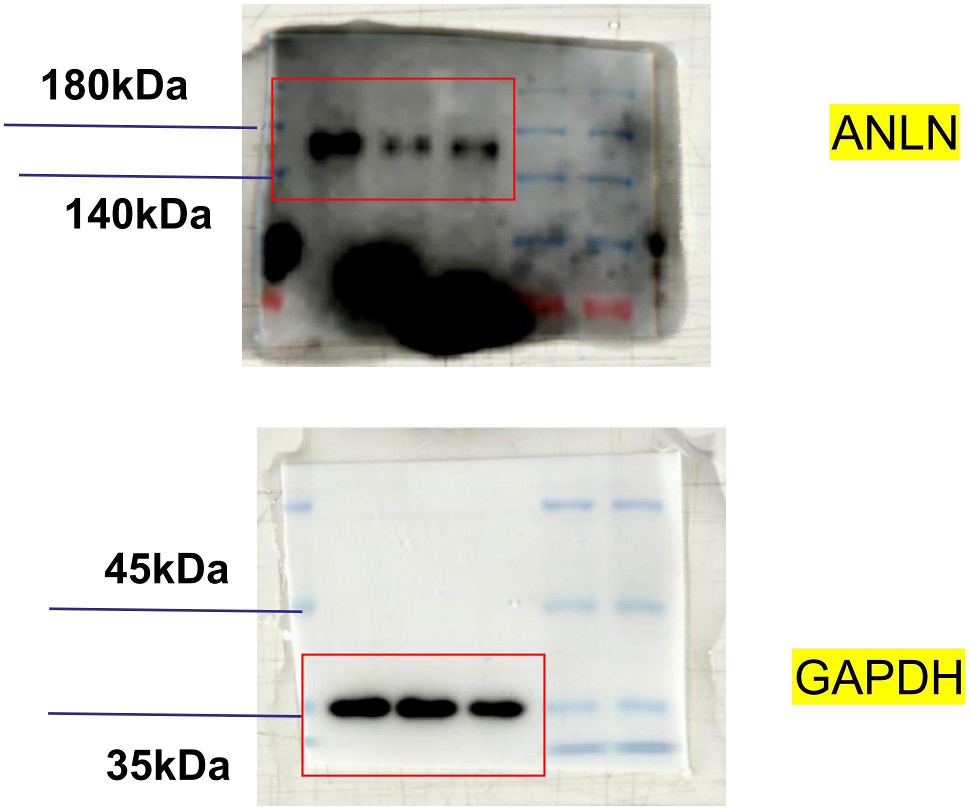

FigS8B

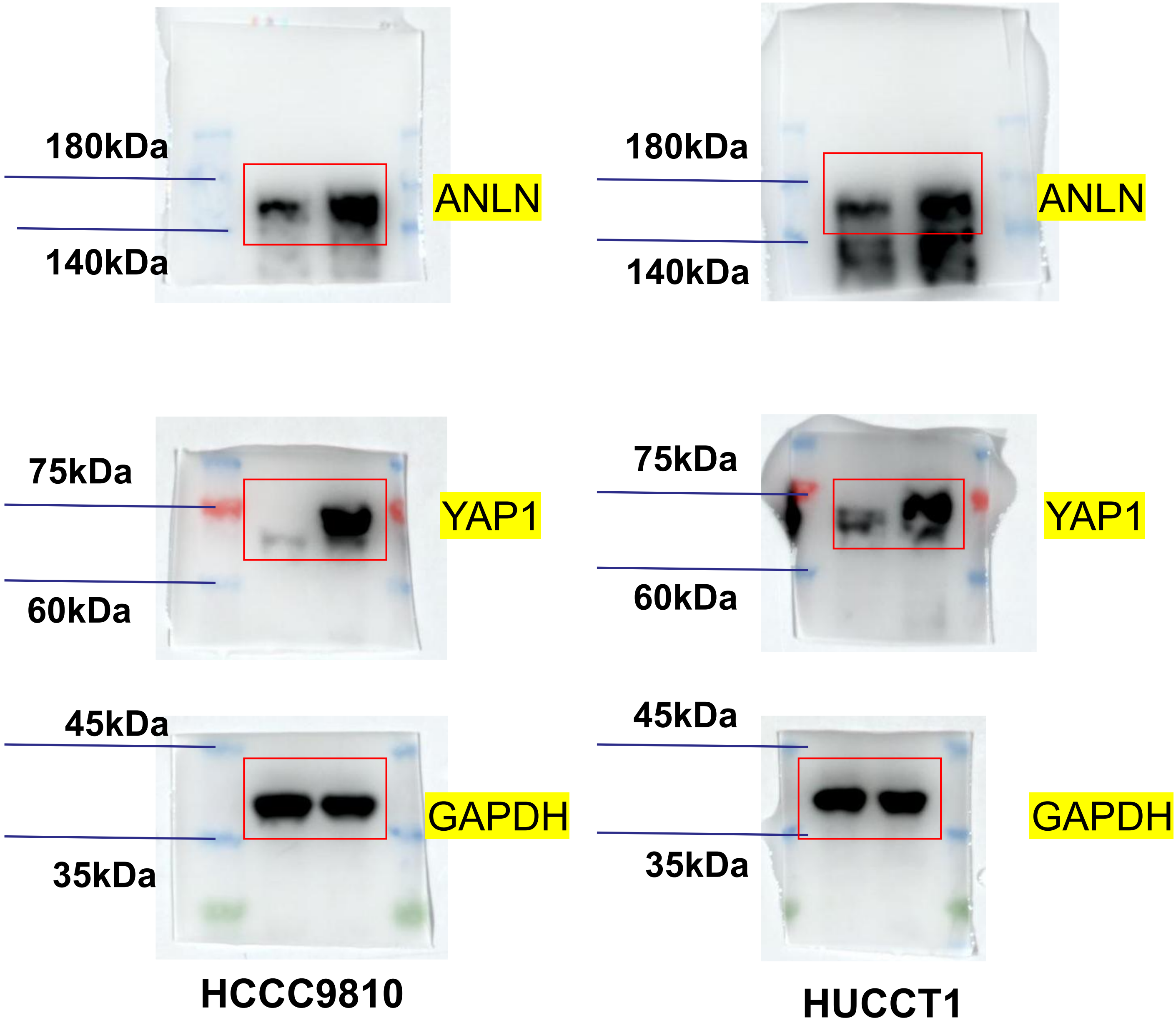
180kDa
140kDa
ANLN
180kDa
140kDa
ANLN
75kDa
60kDa
YAP1
75kDa
60kDa
YAP1
45kDa
35kDa
GAPDH
45kDa
35kDa
GAPDH
HCCC9810
HUCCT1

FigS10A

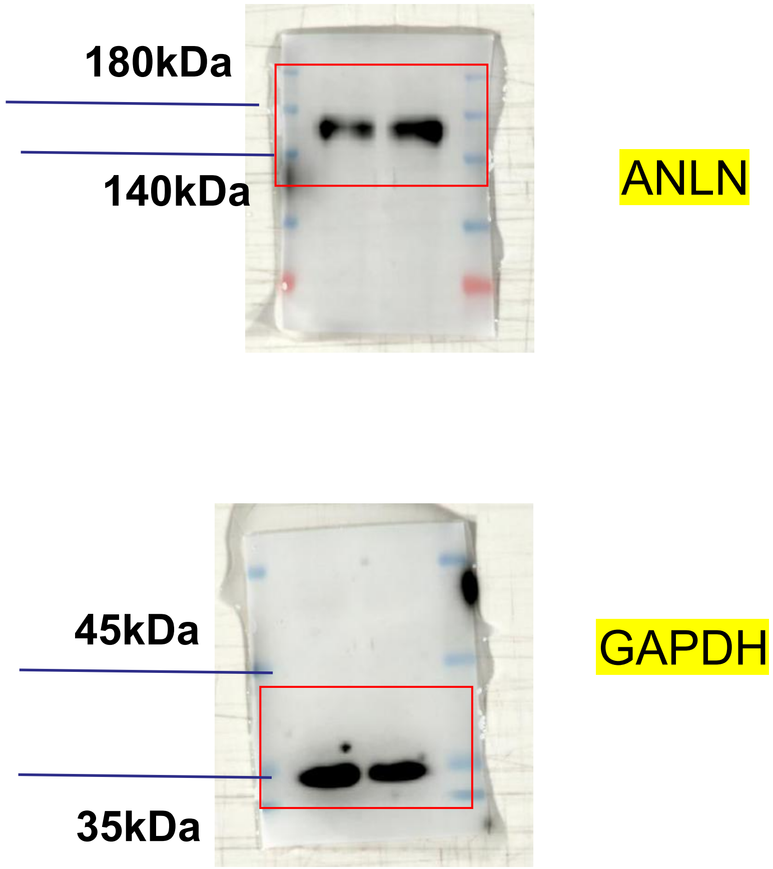

FigS10C

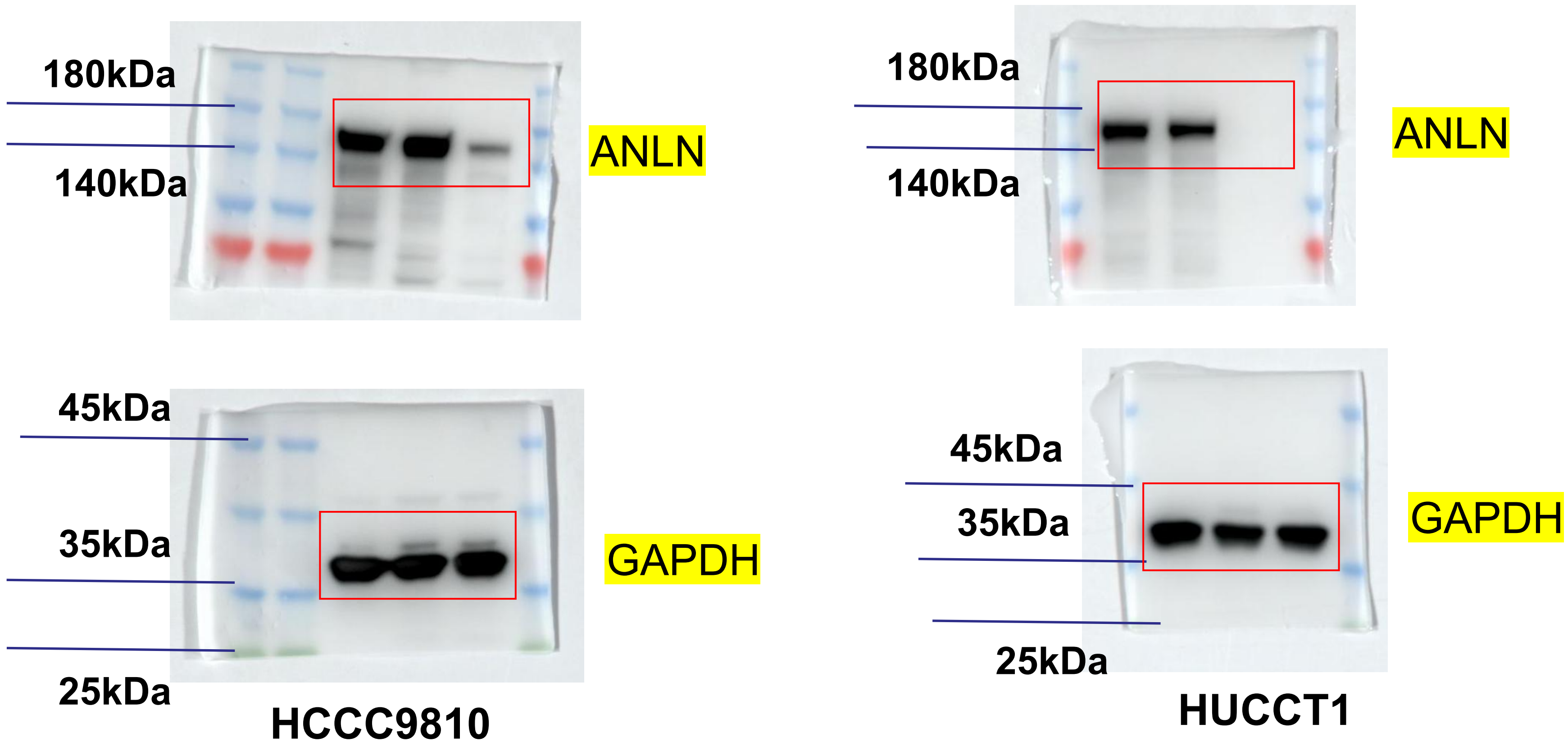
180kDa
140kDa
ANLN
45kDa
35kDa
25kDa
GAPDH
HCCC9810
180kDa
140kDa
ANLN
45kDa
35kDa
25kDa
GAPDH
HUCCT1
